# Differential Inhibition of Anaplerotic Pyruvate Carboxylation and Glutaminolysis-Fueled Anabolism Underlies Distinct Toxicity of Selenium Agents in Human Lung Cancer

**DOI:** 10.3390/metabo13070774

**Published:** 2023-06-21

**Authors:** Teresa W.-M. Fan, Jason Winnike, Ahmad Al-Attar, Alexander C. Belshoff, Pawel K. Lorkiewicz, Jin Lian Tan, Min Wu, Richard M. Higashi, Andrew N. Lane

**Affiliations:** 1 Center for Environmental and Systems Biochemistry, Department Toxicology & Cancer Biology and Markey Cancer Center, University of Kentucky, Lexington, KY 40506, USA; ahmad.al-attar@umassmemorial.org (A.A.-A.); rick.higashi@uky.edu (R.M.H.); andrew.lane@uky.edu (A.N.L.); 2 Department of Chemistry, University of Louisville, Louisville, KY 40202, USA; jwinnike@metabolon.com (J.W.); acbelshoff@gmail.com (A.C.B.); pawel.lorkiewicz@louisville.edu (P.K.L.); 3 Department of Medicine, University of Louisville, Louisville, KY 40202, USA; jinlian.tan@louisville.edu; 4 Seahorse Bioscience, Billerica, MA 01862, USA

**Keywords:** non-small cell lung cancer, selenite, methylseleninic acid, selenomethionine, pyruvate carboxylation, glutaminolysis, chemoprevention, stable isotope resolved metabolomics

## Abstract

Past chemopreventive human trials on dietary selenium supplements produced controversial outcomes. They largely employed selenomethionine (SeM)-based diets. SeM was less toxic than selenite or methylseleninic acid (MSeA) to lung cancer cells. We thus investigated the toxic action of these Se agents in two non-small cell lung cancer (NSCLC) cell lines and ex vivo organotypic cultures (OTC) of NSCLC patient lung tissues. Stable isotope-resolved metabolomics (SIRM) using ^13^C_6_-glucose and ^13^C_5,_^15^N_2_-glutamine tracers with gene knockdowns were employed to examine metabolic dysregulations associated with cell type- and treatment-dependent phenotypic changes. Inhibition of key anaplerotic processes, pyruvate carboxylation (PyC) and glutaminolysis were elicited by exposure to MSeA and selenite but not by SeM. They were accompanied by distinct anabolic dysregulation and reflected cell type-dependent changes in proliferation/death/cell cycle arrest. NSCLC OTC showed similar responses of PyC and/or glutaminolysis to the three agents, which correlated with tissue damages. Altogether, we found differential perturbations in anaplerosis-fueled anabolic pathways to underlie the distinct anti-cancer actions of the three Se agents, which could also explain the failure of SeM-based chemoprevention trials.

## 1. Introduction

Lung cancer is by a large margin the leading cause of death among all cancers for both men and women in the U.S. [1,2]. Most patients arrive at the clinic with advanced lesions due to the silent nature of the disease. Although the five-year survival rate of localized lung cancer is 61.2%, that of advanced stages of the disease drops to 7% f with limited choices of therapeutic regimes and poor prognosis [3,4,5]. Preventive measures and early detection remain the best strategies in the fight against this deadly cancer.

The seminal Nutritional Prevention of Cancer (NPC) trials conducted by Clark et al. [6,7] reported the efficacy of a nutritional supplement with selenized yeast (SeY) in reducing the total incidence and/or mortality of different human cancers, including prostate, lung, and colorectal cancers. These results subsequently inspired numerous laboratory studies [4,8,9,10,11,12,13,14,15,16,17,18,19,20,21,22,23,24,25,26,27,28,29,30] and clinical trials [31,32,33,34,35,36,37] to substantiate the chemopreventive and chemotherapeutic effects of different selenium-containing compounds in human cancers, including lung cancer. Besides SeY, which comprises a mixture of selenium compounds, pure selenoamino acids such as selenomethionine (SeM) have been highly favored in recent clinical trials on human cancers, particularly prostate cancer (e.g., http://clinicaltrials.gov/ct2/show/NCT00736645, http://clinicaltrials.gov/ct2/show/NCT01497431 accessed 28 November 2017, [32,33,34]). However, the large Selenium and Vitamin E Cancer Prevention Trial (SELECT) demonstrated SeM to be ineffective in preventing prostate, lung, or colorectal cancers in healthy recruits [34,35,36,37]. This apparent contradiction to the outcomes of the previous trials could be attributable to the adoption of different doses and/or chemical forms of Se supplements. Many of these rely on the production of anti-cancer Se metabolites. However, little is known of these metabolites in vivo due to their high reactivity and low abundance [38]. Despite the difficult task in speciating the active Se form(s), particularly in human trials, a better understanding of how different Se chemicals perturb cancer cell and tissue functions can help explain the controversy while facilitating the rational design of appropriate doses and forms of Se supplements in future trials.

Several forms of Se compounds including selenite, SeM, methylseleninic acid (MSeA), and methylselenocysteine (CH_3_-SeCys) have been shown to inhibit cancer cell proliferation, induce apoptosis, and attenuate tumor growth and metastasis in mouse xenografts [12,15,17,22,23,26,39,40,41,42,43]. It is clear that the growth inhibitory or anti-metastatic effect of “selenium” on cancer cells or tumor xenografts is highly dependent on the chemical form [17,22,23,38,43,44]. For lung or prostate cancer cells and xenograft tumors, MSeA and selenite are consistently more toxic than SeM [12,22,23,26,45,46,47]. The production of reduced oxygen species (ROS) such as superoxide and hydrogen peroxide induced by these compounds is considered to be the key to their toxicity to cancer cells [24,29,48,49,50]. For example, selenite treatments of cancer cells induced mitochondrial superoxide anion production, which was accompanied by membrane depolarization, permeability transition pore opening, and apoptosis [50,51,52,53]. SeM treatments alone were ineffective but cotreatment with a methionine γ-lyase (methioninase) (which presumably liberates the active methylselenol species) led to similar mitochondrial dysfunctions and apoptosis [54]. MSeA (a stable precursor to methylselenol) treatments have also been shown to elicit ROS-linked apoptosis [24,55].

Despite our understanding of mitochondrial ROS-mediated apoptosis, it is unclear how different Se compounds may impact cellular metabolism to elicit differential inhibition of cell proliferation or other adverse effects, including oxidative stress. By coupling ^13^C tracer studies with metabolomics analysis (i.e., Stable Isotope-Resolved Metabolomics or SIRM), we showed that selenite inhibited several central metabolic pathways including the Krebs cycle in human non-small cell lung cancer (NSCLC) A549 cells [56]. The products of the Krebs cycle (e.g., citrate, Asp, Glu) are the precursors to the synthesis of lipids, nucleic acids, and proteins [57], all of which are required for cell proliferation. Using ^3^H-nucleotides or ^13^C_6_-glucose as tracers, we and others also showed inhibition of RNA and DNA synthesis by selenite [58] or MSeA [59] in A549 cells which is consistent with altered precursor production and inhibition of cell proliferation.

To replenish the Krebs cycle products diverted to macromolecular synthesis, cells utilize two major anaplerotic pathways, i.e., pyruvate carboxylation (PyC) and glutaminolysis, initiated respectively by the enzymes pyruvate carboxylase (PC) and glutaminase (GLS). We have previously shown that PC but not GLS is predominantly upregulated in human lung tumor tissues [60,61]. However, GLS is active and abundant in both cancerous (CA) and adjacent non-cancerous (NC) lung tissues (60 and Fan et al., unpublished data). The kidney isoform of GLS (GLS1) is regulated by the oncogene *MYC* [62] and we have shown that its activity and downstream Krebs cycle-independent pathways is crucial to cancer cell survival and proliferation [63]. We also showed that GLS1 was overexpressed in MYC-driven lung tumors in transgenic mouse models and that inhibition of GLS1 activity led to cancer cell death [64]. These and other previous findings [65,66] underlie the recent interest in targeting GLS1 for cancer therapy [67,68]. Thus, anaplerotic Krebs cycle pathways involving both PC and GLS are important for supporting lung cancer growth. A key question for the variable anti-cancer efficacy of Se agents is if and how such growth-promoting metabolic pathways may be differentially perturbed in lung cancer cells harboring different oncogenic lesions, leading to variable blockades of cell proliferation by these agents.

The SIRM approach that we have developed for the large-scale elucidation of cancer metabolism [59,60,61,63,64,69,70,71,72,73,74,75,76] is well suited for addressing this question in cancer while elucidating the unexpected metabolic dysfunctions induced by different Se agents. SIRM utilizes a combination of nuclear magnetic resonance spectroscopy (NMR) and mass spectrometry (MS) to track the incorporation of stable isotopes such as ^13^C or ^15^N from labeled precursor(s) into various products during metabolic transformations. From the specific labeling patterns of the products, i.e., position (isotopomers) and number (isotopologues) of labeled atoms, a large number of metabolic pathways in different subcellular compartments can be robustly reconstructed, such as glycolysis, pentose phosphate pathway (PPP), the Krebs cycle, pyruvate carboxylation, glutathione biosynthesis, glutaminolysis, nucleotide biosynthesis, and lipid metabolism, to name a few [59,60,63,69,70,71,72,73,74,75,76].

Here we report the use of uniformly ^13^C labeled glucose (^13^C_6_-Glc) or glutamine (^13^C_5_,^15^N_2_-Gln) as tracers coupled with SIRM to track changes in the activity of the Krebs cycle and pathways to lipids and nucleotide synthesis in two lung adenocarcinoma (A549 and H1299) cell lines in response to selenite, MSeA, or SeM treatments. A549 cells harbors *KRAS* mutation but wild type *TP53* while H1299 cells bear an *NRAS* mutation and *TP53* depletion [77,78,79]. We found that the growth and metabolic effects of SeM occurred at nearly 100-fold higher doses than selenite and MSeA and the selenite effect was highly dependent on the types of oncogenic lesions. Different Se agents varied in their effects on the canonical Krebs cycle activity and the two anaplerotic inputs into the Krebs cycle, i.e., PC and glutaminolysis, which led to differential inhibition of lipid and nucleotide biosynthesis. Blockade of these pathways by selenite and MSeA, but not by SeM, related to the effects on cell proliferation but not as well to the metastatic potential. Suppression of PC or GLS1 by shRNA knockdown in A549 and H1299 cells blocked cell proliferation, induced cell death, and arrested cell cycles. More intriguingly, both MSeA and selenite blocked the Krebs cycle and anaplerotic pathways in selected ex vivo organotypic cultures (OTC) of cancerous (CA) lung tissues but had no significant effects in the adjacent non-cancerous (NC) tissues freshly resected from patients with early stages of NSCLC. The patient-derived OTC model closely mimics the natural tumor microenvironment, as it maintains all the original cell complexity and architecture, while providing a direct comparison for the relative toxicity of cancer versus matched non cancer tissues for each agent tested [80,81]. Overall, our study revealed differential metabolic actions of MSeA, selenite, and SeM that could account for their distinct anti-cancer efficacy. These differences in actions could also be related to their distinct chemopreventive efficacy and help explain the failure of the SeM-based trials on lung cancer prevention.

## 2. Materials and Methods

### 2.1. Cell Culturing, Se Treatments, and Metabolite Extraction

A549 and H1299 cells were obtained from ATCC. All cell culture experiments were performed using 10 cm culture plates in a humidified 5% CO_2_/95% air incubator at 37 °C. Culture media consisted of Dulbecco’s Modified Eagle base Medium (DMEM) supplemented with 2 g/L glucose, 4 mM glutamine, 10% FBS, and penicillin (1000 U)-streptomycin (1 mg/mL). Stable isotope tracer experiments used DMEM medium with [^13^C_6_]-glucose or [^13^C_5_,^15^N_2_]-glutamine in place of unlabeled glucose or glutamine, respectively, plus used PBS vehicle (control), 5 µM MSeA, 6.25 µM selenite, or 500 µM SeM as the four treatments. The MSeA treatment medium was renewed at the treatment midpoint. Cells were incubated in each treatment medium for 24 h before metabolic quenching in cold 100% CH_3_CN, followed by extraction in CH_3_CN:H_2_O:CHCl_3_ in a 2:1.5:1 ratio using a modified Folch extraction method [82] to simultaneously yield a polar fraction, a nonpolar fraction, and cell residue. Polar extracts were aliquoted and lyophilized and nonpolar extracts vacuum-dried before analysis for metabolites. Cell residues were extracted for proteins using Western blot sample buffer (62.5 mM Tris + 2% SDS + 1 mM dithiothreitol at pH 6.8) with DNA sheared through an insulin needle. Proteins were denatured at 95 °C for 10 min and quantified using a bicinchoninic acid (BCA) kit according to the vendor’s protocol (Pierce Chemical, 4722 Bronze Way, Dallas, TX, USA). One hundred µL of cell culture media was extracted with 33 µL 40% TCA and after fast centrifugation, the supernatant was lyophilized to dryness overnight.

### 2.2. Ex Vivo Organotypic Tissue Culture (OTC) Experiments

All human specimen collections followed an approved IRB protocol at the University of Louisville. Fresh cancerous (CA) and matched non-cancerous (NC) lung tissues of NSCLC patients were obtained from the operating room immediately after surgical resection. They were thinly sliced (ca. 1 mm thick) by the surgeon as described previously [60] and incubated with gentle rocking for 24 h in the same 4 treatment media as the cell culture experiments above, except for MSeA at 10 µM. The MSeA treatment medium was renewed at the treatment midpoint. At the end of incubation, tissues were harvested by rinsing twice in ice-cold PBS and once in cold nanopore water to remove medium salts. A small piece was cut from the tissue slices for fixing in 4% formalin and the rest cryopreserved in liquid N_2_. Cryopreserved tissues were pulverized into <10 µm particles using a cryo ball mill (SPEX 6770) before extraction using the modified Folch method [82]. Polar extracts were aliquoted and lyophilized before metabolite analysis.

### 2.3. NMR Spectroscopy

A portion of the lyophilized powder was reconstituted in D_2_O containing d_6_-DSS (50 nmoles) before NMR analysis on a 14.1 T Varian Inova spectrometer (Varian Inc., Palo Alto, CA, USA). 1-D ^1^H Presat spectra were recorded at 20 °C with an acquisition time of 2 s, a relaxation delay of 3 s, and 512 transients with the residual HDO signal suppressed by weak rf irradiation. The 1D ^1^H-{^13^C}HSQC spectra were recorded at 20 °C with 1024 transients, an acquisition time of 0.15 s, and a recycle time of 1.5 s. Free induction decays were zero-filled twice and apodized with an unshifted Gaussian function and a line broadening exponential of 0.5 Hz (Presat) or 4 Hz (HSQC) [83]. Compound identification, quantification, and isotope distributions were determined as previously described [83,84].

### 2.4. GC-MS Analysis

A total of 50 µL of 100 µM norleucine as external standard was added to one-tenth of the polar cell or medium extract, with or without 50 µL 40% TCA, respectively, and lyophilized. The resulting powder was derivatized with 50 µL N-(tert-butyldimethylsilyl)-N-methyltrifluoroacetamide:acetonitrile (1:1 *v*/*v*) (MTBSTFA) (Regis Chemical, Morton Grove, IL, USA) and sonicated for 3 h. The derivatized solution was run on a PolarisQ GC-ion trap MSn (ThermoFinnigan, Austin, TX, USA) as previously described [57]. Metabolites were identified and quantified using Xcalibur 3 software (Thermo Scientific, Austin, TX, USA) as previously described [56].

### 2.5. Ion Chromatography-Ultrahigh Resolution Fourier Transform Mass Spectrometry (IC-UHR-FTMS^1^)

Lyophilized cell extracts were freshly reconstituted in 30 μL 18 MΩ water plus 1 µM sodium trimethylsilylpropanesulfonate. Polar extracts (10 µL each) were injected on an IonPac AG11-HC-4 μm guard column (2 × 50 mm) coupled to an IonPac AS11-HC-4 μm RFIC&HPIC (2 × 250 mm) analytical column in a Dionex ICS5000^+^ system (Thermo Scientific). Conditions for chromatographic separations (i.e., KOH gradient), ion suppressor, and desolvation in the heated electrospray were as described previously [85]. Each injection was preceded with a 15 min blank (water) full gradient analysis to clear any carryover contamination, and injections of calibration standard mixtures were before and after each batch of 12 samples to ensure the stability of MS signals. MS data were acquired using the Xcalibur software.

### 2.6. FT-ICR-MS and FT-MS Analysis

For lipid analysis, nonpolar extracts in CHCl_3_:CH_3_OH:butylated hydroxytoluene (2:1:1 mM) were diluted 1:5–10 with methanol and 1 nM reserpine standard. Samples were run on a hybrid linear ion trap-FT-ICR (Finnigan LTQ FT) or an Orbitrap Fusion^TM^ Tribrid^TM^ mass spectrometer (Thermo Electron, Bremen, Germany) equipped with a TriVersa NanoMate ion source (Advion Biosciences, Ithaca, NY, USA), in both positive and negative ionization modes as described previously [86]. For nucleotide analysis, polar fractions were enriched for nucleotides by ion-pairing with hexylamine and cleanup using Pierce C-18 tips (Thermo Fisher Scientific, Waltham, MA, USA) before the analysis in negative ionization mode as described previously [69]. Exact mass data were assigned using an in-house software PREMISE 3.0 (PRecise Exact Mass Isotopologue Search Engine) [86]. ^13^C and/or ^15^N natural abundance correction was performed as described previously [76].

### 2.7. Cell Proliferation Assay

A549 and H1299 cells were seeded in 96-well plates and incubated at 37 °C/5% CO_2_ overnight. Media was removed and replaced with media at 6 different concentrations of MSeA, SeO_3_, or SeM (plus untreated controls), with 6 replicates per concentration, and two time points. After 24 or 48 h, a colorimetric assay (MTT assay) was performed to measure cell viability and proliferation. Measured absorbance was normalized to the untreated control for each of the six replicates. For each time point and selenium compound, a linear regression of normalized absorbance and the logarithm of selenium compound concentration, for 3–6 concentration values, was used to calculate the EC_50_ for each of the six replicates. The six replicates were averaged to obtain the final EC_50_ values, for each of the three selenium compounds, at 24 and 48 h.

### 2.8. Apoptosis/Necrosis, ROS, and Cell Cycle Analyses

These three assays were performed using flow cytometry. Cells were grown to about 70% confluency, trypsinized or lifted using a scraper, washed in PBS, resuspended in appropriate buffers, and counted in trypan blue on a hemocytometer. Approximately 1 × 10^6^ cells were used for each assay.

For measuring apoptosis and necrosis analysis, harvested cells were resuspended in 1X Annexin V binding buffer (BD Pharmingen, San Diego, CA, USA) before the addition of 5 µL Annexin V FITC and 5 µL of propidium iodide (PI), followed by a gentle vortex and incubation in the dark at room temp for 15 min. Prior to acquisition on a BD LSR-II flow cytometer, 400 µL 1X binding buffer was added and analysis was performed within 1 h of staining. Healthy cells stained negative for both Annexin V and PI; apoptotic cells were positive for Annexin V but negative for PI; late apoptotic/early necrotic cells were positive for Annexin V and PI; and necrotic cells were negative for Annexin V but positive for PI.

For ROS detection, the ROS probes DCFH and DHE (Molecular Probes, Waltham, MA, USA) were diluted in PBS to the final concentration of 10 and 5 µM, respectively. Cells were incubated in 1 mL of the probes at 37 °C for 30–45 min along with three controls (unstained or PBS only, DCFH only, and DHE only). After incubation, an extra 1 mL of PBS was added to each tube, mixed well, and centrifuged for 10 min at 1400 rpm. Cells were re-suspended in 200 µL PBS and passed through a 40 µm cell strainer, if necessary, to remove clumps before analysis on a BD LSR-II flow cytometer.

### 2.9. Cell Cycle Analysis

Cell cycle analysis was done by nuclear staining with the DNA binding dye PI for the quantitation of DNA content. Briefly, harvested cell pellets were broken by gentle vortexing and incubated on ice for 15 min. Then, 3 mL of ice-cold 70% EtOH was added slowly while vortexing and cells were incubated for 1 h on ice in the dark, followed by three washes with 1 mL PBS. The cell pellet was resuspended in 250 µL of PI and RNase A in PBS + 0.1% Triton-X at 0.02 and 0.25 mg/mL, respectively, and incubated for 30 min in 37 °C water bath in the dark before measurement on a BD LSR-II within 4 h. Analysis was done using ModFit LT 3.2 software (Verity Software House, Topsham, ME, USA), which uses an algorithm to fit Gaussian curves to each cell cycle phase.

### 2.10. Transwell Migration Assay

Cells in 2D cultures were detached from the plate with 0.25% trypsin for 3 min and collected. They were centrifuged at 1200 rpm for 5 min and the resulting pellet was resuspended in serum free DMEM media at a density of 3 × 10^6^ cells per mL. A total of 100 µL of this cell solution was added to 8 µm pore sized transwell inserts (Becton Dickinson Labware, Franklin Lakes, NJ, USA) which were placed in individual wells of a 24-well plate, each with 600 µL of media containing 10% FBS plus 5 µM MSeA, 6.25 µM selenite, or 500 µM SeM. Cells were allowed to migrate for 6 h. After this time, the tops of the inserts were cleared of unmigrated cells with cotton swabs and the inserts were placed in a 0.5% crystal violet, 25% methanol solution for at least 10 min to fix and stain the cells. Inserts were thoroughly rinsed in RO water and dried overnight. Dye was eluted with 300 µL of a 100 µM sodium citrate in 50% ethanol solution and quantified using a SpectraMax M5 microplate reader at 585 nm.

### 2.11. Enzyme Assays

Cell lysates for enzymatic assays were collected by the trypsin harvest of A549 or H1299 cells seeded on 10 cm plates and treated with 5 μM MSeA, 6.25 μM selenite, or 500 μM SeM for 24 h. Pyruvate dehydrogenase (PDH), lactate dehydrogenase (LDH), NADP-dependent isocitrate dehydrogenase (IDH), and glutaminase (GLS) were assayed from whole cell lysates. These lysates were prepared by resuspending cell pellets in 80 μL 0.1 M Tris, pH 8 with HALT protease inhibitor cocktail (Pierce Biotechnology, Waltham, MA, USA), 1 mM phenylmethylsulfonyl fluoride, 1 mM benzamidine, 2 mM imidazole, and 1.15 mM Na molybdate, followed by lysing through a 28.5-gauge syringe needle.

For the LDH assay, cell lysates were added to a reaction mixture containing 100 mM sodium phosphate, 0.12 mM NADH, 2.3 mM pyruvate, and 0.033% (*w*/*v*) bovine serum albumin, pH 7.5. LDH activity oxidizes NADH to NAD^+^. For the GLS assay, cell lysates were added to a reaction mixture containing 200 mM potassium phosphate, 20 mM glutamine, 5 mM αKG, 0.28 NADH, 55 U/mL glutamate dehydrogenase (GDH), and 0.22 mM EDTA, pH 9. GLS catalyzes the reaction: Gln + H_2_O → Glu + NH_4_^+^ and GDH catalyzes the reaction: NH_4_^+^ + αKG + NADH → Glu + NAD^+^. The enzymatic activity of purified bovine PC (Sigma, St. Louis, MO, USA) was assayed as a coupled reaction with malate dehydrogenase (MDH). PC catalyzes the reaction: pyruvate + HCO_3_^−^ + MgATP → OAA + MgADP + Pi and MDH catalyzes the reaction: OAA + NADH → malate + NAD^+^. The reaction mixture contained 100 mM triethanolamine (TEA) buffer with 5 mM MgSO_4_, 0.1% BSA, 2.5 mM ATP, 18 mM KHCO_3_, 6 mM pyruvate, 0.22 mM NADH, 0.025 mM acetyl CoA, and 25 U/mL MDH, pH 7.8.

In all cases, the consumption of NAD(P)H was monitored by recording the absorbance of the reaction mixture on a SpectraMax M5 microplate reader (Molecular Devices, San Jose, CA, USA) at 340 nm. Initial velocities (mM/min) were determined by taking the negative slope of absorbance versus time (when the rate was linear) and dividing by the extinction coefficient of NAD(P)H (6.22 mM^−1^ cm^−1^) and the path length (0.73 cm). Velocities were converted to milliUnits (mU), which were normalized to protein concentrations as determined by the BCA method.

### 2.12. Oxygen Consumption Analysis

A549 and H1299 cells were plated into a 96 well XF culture plate (Agilent Technologies, Santa Clara, CA, USA) at 10,000 cells per well and incubated at 37 °C/5% CO_2_ overnight. Oxygen consumption rate (OCR) were measured using XF^e^96 analyzer as described previously [87]. Briefly, the cell culture medium was replaced with XF assay medium and incubated at 37 °C for 30 min. Prior to the first compound addition, baseline OCR was measured three times. Compounds were injected at indicated time points denoted by arrows.

### 2.13. shRNA Knockdown Experiments

PC or GLS1 knockdown were done using appropriate lentivirus-based vectors as described previously [88,89]. Briefly, lentivirus was produced in HEK293T cells using psPAX2 and pMD2.G packaging and envelope vectors (Addgene plasmids #12260 #12259, respectively) and pLKO.1-based transfer vectors (Sigma Aldrich, St. Louis, MO, USA). A549 or H1299 cells were transduced with lentivirus plus 8 μg/mL polybrene for 24 h and split to allow for clonal expansion for 48 h. Puromycin at 1 μg/mL was then added to select for stable integrants. Targeting sequences (Mission shRNA, Sigma-Aldrich) used were shGLS1-1 (clone ID NM 014905.2-1441s1c1): 5′-GCACAGACATGGTTGGTATAT-3′; shGLS1-2 (clone ID NM 014905.2-1576s1c1): 5′-GCCCTGAAGCAGTTCGAAATA-3′; shPC54 5′- CCGGGCCCAGTTTATGGTGCAGAATCTCGAGATTCTGCACCATAAACTGGGCTTTTTG-3′; shPC55 5′- CCGGGCCAAGGAGAACAACGTAGATCTCGAGATCTACGTTGTTCTCCTTGGCTTTTTG-3′. Non-targeting control was catalog #SHC016 from Sigma Aldrich.

### 2.14. Reverse Phase Protein Array (RPPA) and Western Blot Analysis

For RPPA assay, protein extracts (at 0.2–0.5 mg/mL) from the cell culture experiments were printed as two drops per spot onto a slide coated with 16 nitrocellulose membrane pads (Grace Bio-Labs, Bend, OR, USA) using a microarray printer (ArrayJet, Ltd., Roslin, UK). As described previously [90], membranes were incubated in FastGreen protein stain and scanned at a 700 nm emission wavelength with InnoScan 710 AL Microarray Scanner (Innopsys, Inc., Carbonne, France) to determine the amount of proteins deposited per sample spot. Slides were then incubated in the blocking buffer (5% FBS in TBST), followed by incubation in a primary antibody in the blocking buffer (see below for vendor and dilution information) against a selected protein for 2 h, washing in TBST, incubation with a fluorescent secondary antibody (LICOR-IRDye 800) at 1:1000 dilution in the blocking buffer for 1 h at 20 °C, washing in TBST, and drying via vacuum suction. Slides were scanned at 800 nm emission wavelength with InnoScan 710 AL. For low abundance proteins, slides were incubated in an appropriate biotinylated secondary antibody (1:15,000 dilution in the blocking buffer) for 1 h at 20 °C, washed in TBST three times at 5 min each before incubation in neutravidin conjugated with DyLight^TM^ 800 (1:8000 dilution in the blocking buffer) (Thermofisher, Waltham, MA, USA) for 30 min at 20 °C, washed three times in TBST at 5 min each, and dried by vacuum suction for scanning at an 800 nm emission wavelength. Fluorescence image analysis of spots was done using Innopsys’s Mapix software v. 9. Background fluorescence for each spot was subtracted from the fluorescence signal for that spot followed by normalization to the FastGreen signal. Normalized signals were averaged across replicates (n = 15–19). The sources of the primary antibodies used are listed in Table 1 below.

### 2.15. Immunohistofluorescence Analysis

Immunohistofluorescence imaging of lung OTC was performed as described previously [91]. Briefly, Formalin-fixed and paraffin-embedded tissue specimens as 4-μm thick tissue sections on microslides were deparaffinized, rehydrated, subjected to antigen retrieval in 10 mM sodium citrate buffer (pH 6.0), and permeabilized in methanol at −20 °C for 10 min before blocking with 10% rabbit serum, 3% BSA, and 0.3% Triton X-100 in PBS at room temperature for 1 h. Slides were then incubated in primary antibodies at 4 °C overnight and washed in PBS before incubation in the fluorescent conjugated secondary antibody in the dark at room temperature for 1 h and washed in PBS. The primary antibody against PCNA was obtained from Cell Signaling Technology (XP Rabbit mAb D3H8P at 1:800 dilution) and the fluorescent conjugated secondary antibody (goat anti-rabbit IgG (H + L) Alexa Fluor 488 at 1:100 dilution) was from Thermo Fisher Scientific. Stained slides were mounted in the ProLong Gold Antifade Mountant with DAPI (P-36931, Thermo Fisher Scientific) and fluorescent images were acquired using a laser scanning confocal microscope Olympus FluoView FV1000 with a 20X objective. The number of total and PCNA-positive cells were counted in 2–4 representative regions for each fluorescent image, from which the fraction of PCNA-positive cells was calculated.

### 2.16. Statistical Analyses

Means of control and treatment pairs were compared using the unpaired Students’ two-tailed t-test either with Excel (v 16), or with GraphPad Prism 9 (Dotmatics, Boston, MA, USA). For multiple comparisons, we used the *Q*-value approach to control for the false discovery rate, as described in the Supplementary Tables. A value of *Q* < 0.05 was taken as statistically different. All *Q* values are provided in the Supplementary Tables.

## 3. Results

### 3.1. Lung Cancer Cell Proliferation Is Inhibited by Selenite, MSeA, and SeM but Their EC_50_ Differs Widely and Is Cell Line-Dependent

The effect of MSeA, selenite (SeO_3_), and SeM on cell proliferation were determined in 2D cultures of human A549 and H1299 adenocarcinoma lung cancer cells cultured in standard conditions for 24 and 48 h as described in the Methods (Supplemental Figure S1). MSeA and selenite but not SeM elicited morphological changes in A549 and H1299 cells although selenite’s effect was less evident in both cell lines after 48 h of treatment (Figure S1A). More importantly, MSeA and selenite had much lower EC_50′_s (3 to 80 µM) compared to SeM (unmeasurable at 24 h and 2–500 µM at 48 h) in A549 and H1299 cells after 24 and 48 h of treatments. At low µM levels, SeM actually stimulated A549 cell growth. The EC_50′_s at 24 and 48 h of MSeA treatment (4.8 and 3.7 µM) were comparable for H1299 and A549 cells but the EC_50′_s at 24 and 48 h of selenite treatment in H1299 cells were 6 to 10-fold higher than those in A549 cells. For the MSeA treatment, there was a sharper decrease in EC_50_ from 24 to 48 h for A549 than for H1299 cells, while a longer exposure to selenite resulted in a greater drop in EC_50_ for H1299 than for A549 cells. Consistent with the effect on cell proliferation, the protein weights decreased accordingly in response to 24 h of 5 µM MSeA, 6.25 µM selenite, or 500 µM SeM treatment for the two cell lines (Figure S1C). Thus, the three Se agents differed in their capacity to inhibit lung cancer cell proliferation, which is consistent with other reports [22,23]. Our study also showed that the inhibitory action of SeO_3_ varied with *TP53* status. Both A549 and H1299 cells are of adenocarcinoma origin, but A549 has a wild type *TP53* [22] while H1299 is *TP53*-null [88]. This difference may underlie their differential sensitivity to selenite since p53 has been reported to mediate selenite-induced cytotoxicity [51,89,92]. In contrast, the MSeA effect on proliferation was independent of the p53 status, as the efficacy of MSeA was comparable between A549 and H1299 cells (Figure S1B). MSeA-induced growth arrest was also shown to be independent of p53 in human prostate cancer cells [8].

### 3.2. Selenite, MSeA, and SeM Differentially Induce ROS/Apoptosis and Inhibit Metastatic Potential in Lung Cancer Cells

We then examined how the three Se agents differed in their ability to induce apoptosis, production of reactive oxygen species (ROS), cell cycle arrests, and cell migration in A549 and H1299 cells after 48 h of exposure. As shown in Figure S2A and Table S1**,** >60% of MSeA-treated A549 cells underwent apoptosis while only a small fraction of SeO_3_ or SeM-treated cells were apoptotic. The necrotic responses of A549 cells induced by the three agents followed a similar trend, albeit to a much lower extent. The same trend was also evident for H1299 cells in response to MSeA, SeO_3_, or SeM.

We also found that the extent of apoptosis did not correlate with the ROS production induced by the three Se agents in the two cell lines, e.g., MSeA elicited more apoptosis but much less ROS production than SeO_3_ in A549 cells (Figure S2B). ROS production in H1299 cells was slightly enhanced by SeM but suppressed by MSeA or SeO_3_, which again contrasted with the apoptotic responses.

In terms of the cell cycle responses, MSeA was most effective in arresting the cell cycle at the G1 phase, followed by SeM in A549 and H1299 cells. Selenite had a minor effect on the cell cycle at the G2/M phase in A549 cells and arrested H1299 cells at the S phase (Figure S2C). These cell cycle effects of MSeA and SeO_3_ are also evident in other cell types [44].

Furthermore, the effect of the three Se treatments on metastatic potential (assessed by cell migration assays) of A549 and H1299 cells varied, as shown in Figure S2D. MSeA at 5 µM was effective in blocking the migration of both cell lines, while SeO_3_ at 6.25 µM was either less effective (H1299) or ineffective (A549), and SeM at 1 mM had either no effect (H1299) or a stimulatory effect (A549) (Figure S2C).

To see if and how the differential phenotypic changes described above are accompanied by metabolic perturbations, we treated A549 or H1299 cells with the three Se agents in the presence of ^13^C_6_-Glc or ^13^C_5_,^15^N_2_-Gln for 24 h and analyzed the metabolite profiles plus their isotopic labeling patterns in treatment media and cell extracts by NMR, GC-MS, and FT-ICR-MS.

### 3.3. Selenite and MSeA Perturb Glycolysis and Major Nutrient Consumption in Lung Cancer Cells but These Effects Cannot Account for the Altered Phenotypes

**Figure 1:**
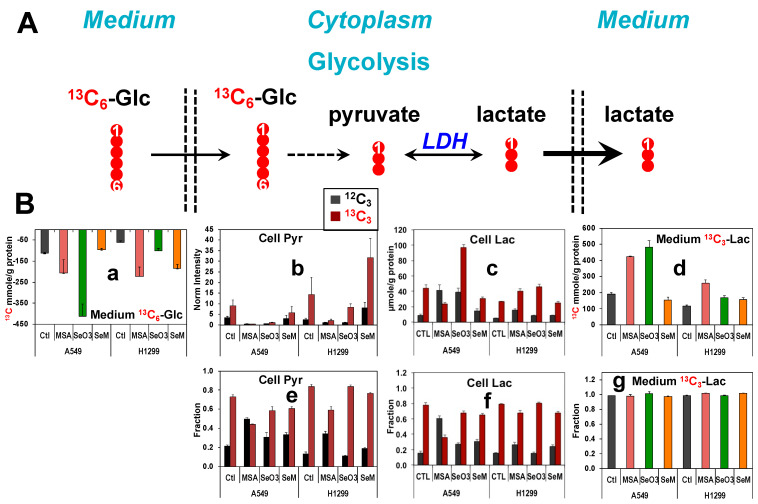
Distinct capacity of MSeA, selenite, and SeM to stimulate glycolysis in A549 and H1299 cells. A549 or H1299 cells were treated with 5 µM MSeA, 6.25 µM selenite, or 500 µM SeM for 24 h with ^13^C_6_-Glc (n = 3) as tracer, as described in Methods. Medium lactate (Lac, **B**(**d**,**g**)) was analyzed by ^1^H NMR, cell pyruvate (Pyr) by QDA-FTMS (**B**(**b**,**e**)), and cell Lac (**B**(**c**,**f**)) determined by GC-MS. Uptake of ^13^C_6_-Glc (**B**(**a**)) and subsequent glycolysis was traced in (**A**) with ● as ^13^C LDH: lactate dehydrogenase. See Table S2 for statistical analysis.

Figure 1 shows that 5 µM MSeA or 6.25 µM selenite stimulated glucose metabolism via glycolysis (Figure 1A) in A549 cells, as evidenced by the increase in both ^13^C_6_-Glc consumption (Figure 1B(a), a measure of glucose uptake) and the release of the glycolytic product ^13^C_3_-lactate (Lac) into the medium (Figure 1B(d)). SeM at 500 µM had little effect on ^13^C_6_-Glc consumption or ^13^C_3_-lactate production by A549 cells. These results indicate glycolytic activation by MSeA and selenite but not by SeM. It should be noted that the altered glycolytic activity cannot be deduced from the level of the intracellular glycolytic products such as ^13^C_3_-pyruvate (Figure 1B(b)) or -lactate (Figure 1B(c)), which has been a common practice. This is because ^13^C_3_-pyruvate or ^13^C_3_-lactate levels in cells and media showed different response trends, e.g., opposite for the MSeA treatment. The medium but not intracellular lactate reflected the glycolytic capacity as it was the predominant pool of lactate produced from glucose (ca. > three orders of magnitude higher in level than the cell lactate). In addition, the fractional enrichment of the ^13^C_3_-lactate medium was essentially 100% (Figure 1B(g)), which indicates that all the lactate medium was derived from ^13^C_6_-Glc. In contrast, the fractional enrichment for ^13^C_3_-pyruvate and -lactate in cells was less than 100% (Figure 1B(e,f)), which suggests a contribution of non-glucose-derived lactate (^12^C_3_ or unlabeled isotopologue) to the total cellular pool. Together, they suggest intracellular compartmentation of two lactate pools, Pool 1 is derived from glucose and primarily exported to the medium while Pool 2 was generated from non-glucose substrates and better retained. MSeA or selenite increased the level and fractional enrichment of non-glucose derived (unlabeled) lactate whereas SeM had only a minor effect in A549 cells (^12^C_3_, Figure 1B(c,f)). These data point to an enhanced lactate production from non-glucose source(s) and possibly an attenuated ^13^C-lactate production from glycolysis. We can rule out the latter as the lactate medium data (Figure 1B(d)) indicates the opposite trend in response to MSeA or selenite.

For H1299 cells, MSeA and SeM showed a similar effect on glucose consumption and glycolytic activity as A549 cells (Figure 1B(a,d)). However, selenite’s effect on glucose consumption and glycolysis in H1299 cells was much less significant relative to that for A549 cells. Suffice to say, activation of glycolysis by MSeA or selenite is inconsistent with their growth inhibitory effects observed in Figure S1.

### 3.4. MSeA and Selenite Block the Krebs Cycle Activity in Lung Cancer Cells While SeM Does Not, Which May Underlie Their Differential Effect on Proliferation

**Figure 2:**
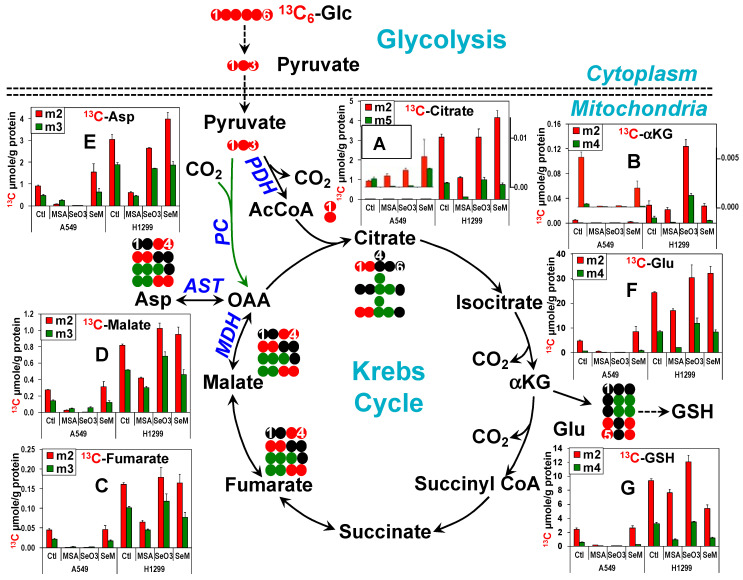
Distinct capacity of MSeA, selenite, and SeM in blocking glucose-fueled Krebs cycle in A549 and H1299 cells. The polar extracts in Figure 1 were analyzed by IC-FTMS for Krebs cycle metabolites. ●, ● track ^13^C derived from ^13^C_6_-Glc via the pyruvate dehydrogenase (PDH) and/or pyruvate carboxylase (PC)-initiated Krebs cycle activity, respectively. Black circle represent ^12^C. (**A**–**G**) normalized metabolite levels. The ^13^C labeled species displayed results from the first cycle. m2–m4 denotes ^13^C_2–4_ isotopologues. αKG: α-ketoglutarate; GSH: glutathione; AST: aspartate aminotransferase; MDH: malate dehydrogenase. See Table S3 for statistical analysis.

Further tracing of ^13^C from ^13^C_6_-Glc into the Krebs cycle in A549 cells showed a remarkable inhibition of the cycle activity by both selenite and MSeA, as shown in Figure 2 (protein-normalized abundance) and Figure S3 (fractional enrichment). Protein normalization is necessary to correct for the influence of differential cell mass on total metabolite abundance, while fractional enrichment data are independent of cell mass differences. MSeA at 5 µM reduced the oxidation of glucose-derived pyruvate in the Krebs cycle, initiated by both pyruvate dehydrogenase (● PDH) and anaplerotic pyruvate carboxylase (● PC) reactions. This was evidenced respectively by the reduced level (Figure 2A–E) and fractional enrichment (Figure S3A–E) of ^13^C_2_-/^13^C_5_-citrate, ^13^C_2_-/^13^C_3_-fumarate, -malate, and -Asp, as well as ^13^C_4_-Glu and glutathione (GSH) (Figure 2 and Figure S3F,G) in A549 and H1299 cells. However, 500 µM SeM had only a relatively minor effect on the ^13^C labeling patterns of these metabolites in A549 and H1299 cells, except for the decrease in level but increase in fractional enrichment of ^13^C_2_-GSH (m2) in H1299 cells. This SeM effect on GSH points to increased GSH utilization by H1299 cells presumably for anti-oxidation. Although selenite at 6.25 µM greatly attenuated the Krebs cycle activity as in the case for MSeA in A549 cells, it failed to do so in H1299 cells. These effects on the Krebs cycle correlated well with those on cell proliferation (Figure S1B). It should be noted that a substantial fraction of the citrate (and downstream metabolites) pool remained unlabeled even after 24 h (cf. Figure S3), indicating precursor source(s) other than glucose, such as glutamine (see Section 3.6). It should also be noted that many Krebs cycle intermediates also occur in the cytoplasm as separate pools, which complicates quantitative metabolic modeling [90,93].

### 3.5. Selenite, MSeA, and SeM Differentially Block O_2_ Consumption and Mitochondrial Respiratory Capacity in Lung Cancer Cells

The differential perturbations of the Krebs cycle activity by MSeA, selenite, and SeM in A549 and H1299 cells were related to the changes of the O_2_ consumption rate (OCR), as shown in Figure S4. MSeA and selenite, but not SeM, inhibited OCR in A549 cells (Figure S4A), while OCR blockade was induced only by MSeA in H1299 cells (Figure S4B). When the OCR was perturbed by oligomycin (Oligo) along with MSeA or selenite, we saw a decreased response for both agents in A549 cells (Figure S4C), while this response was evident only for MSeA in H1299 cells (Figure S4D). The oligo-induced reduction in OCR reflects the cell’s capacity for ATP production. Thus, ATP production was attenuated by both MSeA and selenite in A549 cells but only by MSeA in H1299 cells. Likewise, we observed a decreased OCR response to the uncoupler FCCP induced by MSeA or selenite in A549 (Figure S4C), but not in H1299 cells (Figure S4D). As the FCCP-elicited increase in OCR tracks the mitochondrial electron transport chain (ETC) activity, the observed perturbations suggest a blockade of the ETC by MSeA or selenite in A549 but not in H1299 cells, which can in turn lead to reduced Krebs cycle activity or vice versa. It should be noted that blocked ETC compromises the activity of mitochondrial dihydroorotase dehydrogenase (DHODH), a key enzyme in pyrimidine biosynthesis.

To determine how the mitochondrial respiration decreased, we probed the response in the expression of a key ETC component NADH:ubiquinone oxidoreductase core subunit S1 (NDUFS1) and ATP synthase F0 Subunit 8 (MTATP8) to the three treatments. We found that NDUFS1 (a) and MTATP8 (b) were suppressed by selenite in A549 but not in H1299 cells (Figure S5B), which could account for the differential effect of selenite on the ETC activity (Figure S4) in the two cell lines. Similarly, suppressed NDUFS1 and MTATP8 by MSeA were related to a reduced OCR and ATP production in both cell lines. Since mitochondrially encoded MTATP8 is a target gene of the master mitochondrial transcription factor 1 (TFAM), we also examined TFAM protein expression (Figure S5B(c)) and found its change in pattern to correlate with that of MTATP8 in selenite and MSeA-treated A549 and H1299 cells. Thus, it is plausible that MSeA and selenite block mitochondrial respiration by down-regulating TFAM and thus mitochondrial biogenesis [94].

### 3.6. Selenite, MSeA, and SeM Differentially Block Glutaminolysis in Lung Cancer Cells

**Figure 3:**
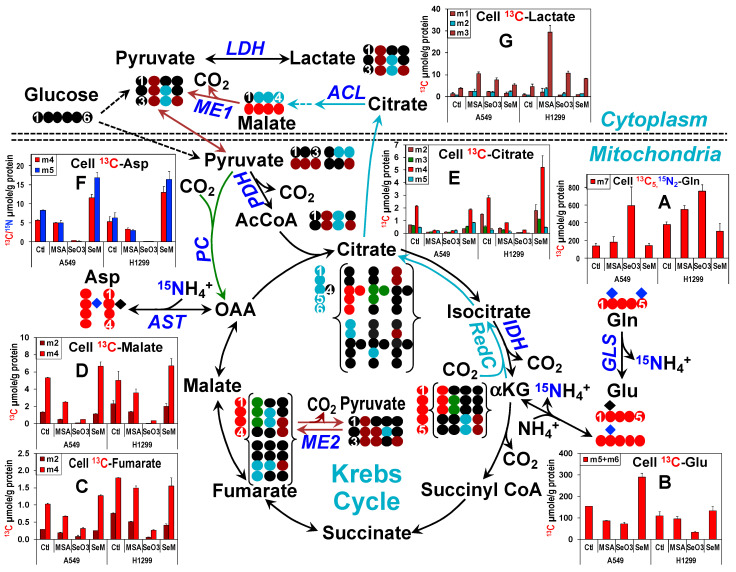
Distinct capacity of MSeA, selenite, and SeM in blocking glutaminolysis in A549 and H1299 cells. A549 or H1299 cells were treated with 5 µM MSeA, 6.25 µM selenite, or 500 µM SeM for 24 h with ^13^C_5_,^15^N_2_-Gln (n = 2) as tracer, as described in Methods. Polar metabolites were analyzed by GC-MS (**A**–**G**). Pathways shown track ^12^C (●), ^14^N (◆), ^13^C from glutaminolysis + PDH-initiated Krebs cycle (●), ^13^C from glutaminolysis + Krebs cycle + ME (●)/ME + PC (●) or glutaminolysis + reductive carboxylation (RedC, ●), and ^15^N (◆) from glutaminolysis/transamination; not all expected ^13^C and/or ^15^N labeled products are shown. The color bars (■, ■, ■, ■) denote the ^13^C isotopologues related to the labeled products in the pathway scheme. m1-6: ^13^C_1-6_ isotopologues; GLS: glutaminase; IDH: isocitrate dehydrogenase; ME: malic enzyme; ACL: ATP-citrate lyase; LDH, PDH, PC, AST: abbreviations as in Figure 1 and Figure 2. See Table S7 for statistical analysis.

We recently showed that selenite blocked glutaminolysis by suppressing GLS1 expression, which can partially account for its toxicity in A549 cells [91]. To determine whether glutaminolysis is also inhibited by MSeA or SeM, we compared ^13^C_5_,^15^N_2_-Gln metabolism in A549 and H1299 cells after 24 h of the three Se treatments. As previously observed, intracellular ^13^C_5_,^15^N_2_-Gln accumulated (A) while two of the most direct GLS products (^13^C_5_,^15^N_1_-Glu or m6 plus ^13^C_5_-Glu^14^N_1_ or m5 derived from ^13^C_5_-αKG, (B)) decreased in selenite-treated A549 cells (Figure 3). MSeA also inhibited the Gln to Glu conversion but to a lesser extent than selenite while SeM showed an opposite effect in A549 cells. H1299 cells displayed a similar response to selenite and SeM on Gln deamidation to Glu but the response to MSeA was negligible.

Despite the large changes in levels, the ^13^C fractional enrichment in m5 + m6 of Glu remained constant under all treatments (Figure S6B), which suggests that the turnover of ^13^C labeled and unlabeled Glu was comparable. We then assayed the mitochondria-associated GLS activity in cell lysates to compare with the SIRM-based *in-cell* assays for GLS activity. Figure S5A(b) showed respectively inhibition and activation of GLS activity by selenite and SeM in A549 cell lysates, which correlated with the changes in the *in-cell* assays. However, the lack of MSeA effect on the GLS activity in A549 cell lysates or of selenite effect on that in H1299 cell lysates contrasted with the significant depletion of ^13^C-Glu in these cells. These results suggest a blockade in GLS-independent glutaminolysis.

To assess glutamine contributions to the Krebs cycle, we further tracked the metabolism of ^13^C_5_,^15^N_1_-Glu or ^13^C_5_-Glu in the canonical Krebs cycle via the formation of ^13^C_4_-fumarate (tracked by ●) (Figure 3C), -malate (Figure 3D), -citrate (Figure 3E), and -Asp (Figure 3F) or in the non-canonical reductive carboxylation pathway [95,96] (RedC tracked by ●) via ^13^C_5_-citrate formation (Figure 3E). Subsequent transformations of ^13^C_4_/^13^C_5_-citrate via the cytoplasmic citrate lyase (ACL)-malic enzyme 1 (ME1, tracked by ●) pathway produces ^13^C_2_/^13^C_3_-pyruvate while the Krebs cycle + the reversible mitochondrial ME2 reactions generate ^13^C_1_ to ^13^C_3_-pyruvate. All ^13^C labeled pyruvate species can reenter the Krebs cycle via the PDH or PC reaction (tracked by ●) to form ^13^C_1_/^13^C_2_/^13^C_3_-citrate, -fumarate, and -malate. ^13^C_5_-citrate can also be formed by condensing ^13^C_3_-citrate with ^13^C_2_-acetyl CoA. Changes in the level of the ^13^C_4_ (m4) species of these metabolites in response to the three Se agents (■ Figure 3) largely tracked those of the Glu precursor. However, the fractional enrichment for these metabolites was attenuated by selenite and MSeA in A549 cells and by selenite in H1299 cells without corresponding changes in the Glu precursor **(■** Figure S6). These data suggest a blockade of the Krebs cycle especially by selenite and to a lesser extent by MSeA in addition to glutaminolysis. Also noted was the reduced enrichment of ^13^C_3_-citrate by MSeA and selenite in A549 and by MSeA in H1299 cells (❚, Figure 3E), which signifies attenuated anaplerotic PC, as was observed in the ^13^C_6_-Glc tracer study (Figure 2). Dilution of ^13^C labeling in pyruvate by glucose-derived unlabeled pyruvate was not considered to be a significant contributing factor to the reduced ^13^C enrichment in citrate. This is because the abundance of glucose-derived pyruvate was greatly diminished by MSeA or selenite treatment (cf. Figure 1B(b)). We further saw that the ^13^C_2_ isotopologues of citrate, fumarate, and malate (❚) did not accumulate, reflecting glutaminolytic activation by SeM in A549 cells, while they were depleted in response to MSeA in H1299 cells, which is unexpected from the lack of glutaminolytic inhibition by MSeA. As these species were formed from the ME reactions, inhibition of ME1 and/or ME2 by SeM in A549 or by MSeA in H1299 cells could account for these changes. Moreover, we noted an enhanced fractional enrichment of ^13^C_5_-citrate by the three Se agents in A549 cells (❚, Figure S6E), which may result from an increase in RedC relative to the forward Krebs cycle activity.

The block in pyruvate metabolism via the Krebs cycle by MSeA or selenite diverted Gln-derived pyruvate to lactate production, as evidenced by the buildup of ^13^C_1-3_-lactate in MSeA or selenite-treated A549 and H1299 cells (Figure 3G). The increase in level and fractional enrichment of ^13^C_2_-lactate (❚) is consistent with enhanced RedC in A549 cells under all three Se treatments. Although ^13^C_2_-pyruvate and thus ^13^C_2_-lactate can also be produced via the mitochondrial ME2 exchange reaction (●), this would involve the formation of ^13^C_2_-malate, which displayed different labeling patterns from ^13^C_2_-lactate. This result suggests that the ME2-derived ^13^C_2_-lactate did not contribute significantly to the total pool.

Altogether, the ^13^C_6_-Glc and ^13^C_5_,^15^N_2_-Gln tracing data indicate that selenite and MSeA but not SeM block the Krebs cycle activity via the canonical and anaplerotic pathways, which is correlated with their differential effect on proliferation (Figure S1B,C). In addition, inhibition of GLS-independent glutaminolysis could contribute to growth inhibition by MSeA in A549 and by selenite in H1299 cells.

### 3.7. Inhibition of PC Activity and Suppression of GLS1 Expression Respectively Contribute to Reduced Krebs Cycle Activity Induced by MSeA and Selenite

Since the two key anaplerotic processes initiated by PyC and glutaminolysis were blocked by MSeA and selenite, we investigated how this blockade was mediated. We probed the protein expression of PC and the three isoforms of GLS by Western Blot (WB) and/or Reverse Phase Protein Array (RPPA). As shown in Figure S5B(d), PC expression was suppressed by selenite in A549 but not in H1299 cells. PC was not significantly suppressed by SeM or MSeA in A549 and H1299 cells. The selenite-induced PC suppression accounted for reduced PyC deduced from the SIRM data (Figure 2 and Figure 3), but the MSeA-induced effect on PC in A549 cells is inconsistent with the substantial block of PyC based on the SIRM data. The assay of purified bovine PC activity revealed a direct inhibition of PC activity by MSeA but not by selenite or SeM (Figure S5A(c)). This MSeA inhibition of PC may be mediated via thiol modifications as the effect required preincubation of MSeA (5 µM) with the enzyme (2.5 mU) in the presence of GSH (13 µM). PC’s catalytic action may involve a cysteine residue [97] and GSH can mediate the reaction of this residue with MSeA. This is analogous to a report on the inhibition of purified protein kinase C by MSeA [98]. MSeA is also known to catalyze modifications of reactive thiol groups in several other cellular proteins [99].

**Figure 4:**
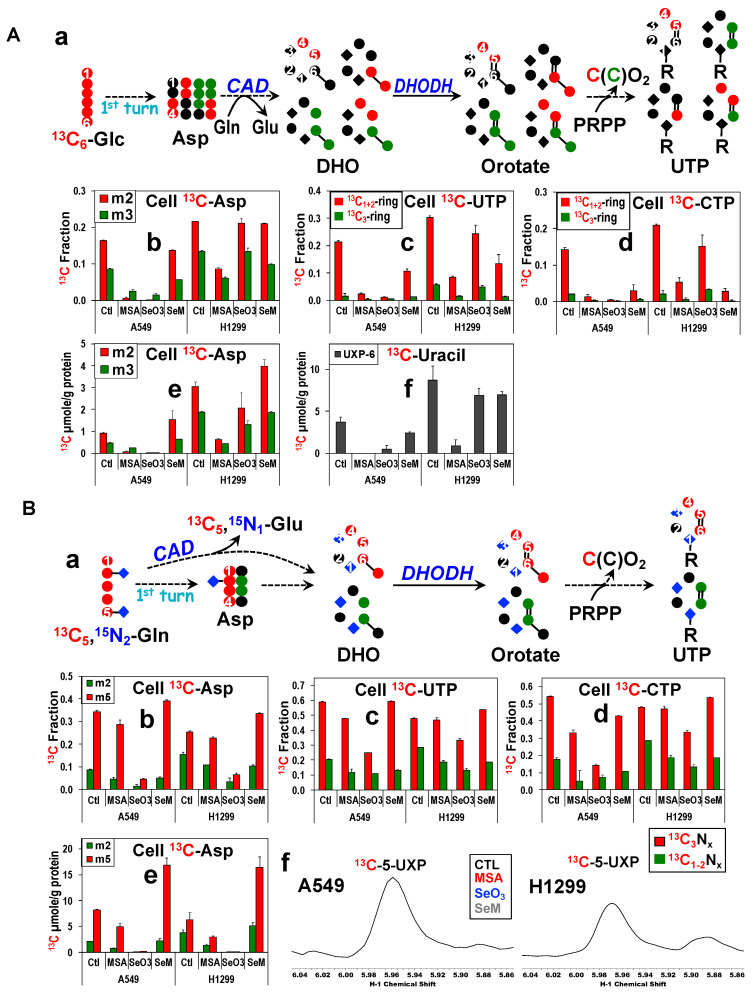
Distinct capacity of MSeA, selenite, and SeM in blocking pyrimidine synthesis from glucose or Gln in A549 and H1299 cells. The same polar extracts in Figure 2 and Figure 3 were analyzed by GC-MS (Asp), IC-FTMS (UTP, CTP), or 1D HSQC (uracil in (**A**(**f**)); UXP in (**B**(**f**))). Data for the ^13^C_6_-Glc (n = 3) and ^13^C_5_,^15^N_2_-Gln (n = 2) tracer experiments are shown in (**A**,**B**), respectively. ●,●,● in (**A**(**a**)) track carbon ^12^C, ^13^C from PDH or PC-initiated Krebs cycle reactions as in Figure 2; ●, ●, ●/●, ◆ in (**B**(**a**)) denote ^12^C, ^13^C from glutaminolysis + Krebs cycle, ^13^C from glutaminolysis + Krebs cycle + ME ± PC ± RedC, ^15^N, as in Figure 3. ■ in (**A**(**b**,**e**)) and (**B**(**b**,**e**)): total ^13^C; ■, ■ in (**A**(**b**–**e**),**B**(**b**–**e**)): ^13^C in Asp and uracil/cytosine ring derived from PDH or PC-initiated Krebs cycle activity, respectively. Nx in B-c,d: ^15^N_0-3_; DHO: dihydroorotate; CAD: carbamoyl phosphate synthetase 2, aspartate transcarbamylase, and dihydroorotase; DHODH: dihydroorotate dehydrogenase. See Table S8 for statistical analysis.

Analysis of GLS1 (KGA/GAC) (Figure S5B(e)) and GLS2 (Figure S5B(g)) by RPPA as well as GLS1′s splice variant GAC (Figure S5B(f)) by WB showed largely similar cell type and treatment dependence (Figure S3B). Notably, mitochondrial GAC [92] was substantially suppressed by selenite in both cell lines but GAC was slightly enhanced by SeM in A549 cells, which correlated with the effects of selenite and SeM on the mitochondrial GLS activity (Figure S5A(b)) and *in-cell* glutaminolysis activity (Figure 3). The lack of inhibition in the GLS activity by MSeA in A549 and H1299 cells and by SeM in H1299 cells could also be related to the response patterns of GAC expression. Thus, altered GAC expression (Figure S5B(f)) accounted for the changes in total GLS activity and thus general changes of *in-cell* glutaminolysis (Figure 3) in both cell lines. However, the lack of GAC/GLS1 suppression or minor GLS2 suppression by MSeA does not explain the reduced conversion of labeled Gln to Glu without the buildup of the former in A549 cells (Figure 3A,B), which points to the contribution of compromised GLS-independent glutaminolysis, such as that occurring in pyrimidine (Figure 4) and purine (Figure S7B) biosynthesis, described below.

### 3.8. MSeA Inhibition of PC and Selenite Suppression of GAC/PC/CAD Lead to Reduced Pyrimidine Nucleotide Synthesis

One of the Krebs cycle products, Asp, is a requisite precursor to the synthesis of pyrimidine rings. We thus asked whether MSeA or selenite can block de novo synthesis of pyrimidine nucleotides from the Glc Figure 4A(a) or Gln tracer Figure 4B(a) by inhibiting the replenishment of Asp via reduced anaplerotic PC, ME, and/or GLS activity. We tracked the ^13^C isotopomers of Asp, uracil, and cytosine that are produced through PDH (●)- and PC (●)-initiated Krebs cycle activity in Figure 4A(a) and those produced through glutaminolysis (●) and ME/PC (●)-initiated Krebs cycle activity in Figure 4B(a). We used FT-ICR-MS to determine the incorporation of ^13^C from ^13^C_6_-Glc or ^13^C_5_,^15^N_2_-Gln into the uracil ring of UTP and the cytosine ring of CTP [85].

First, we noted that Gln was the preferred carbon source for Asp and pyrimidine biosynthesis in both cell lines, as evidenced by the higher ^13^C fractional enrichment in these metabolites derived from ^13^C_5_,^15^N_2_-Gln than those from ^13^C_6_-Glc (Figure 4A vs. Figure 4B). This is consistent with our previous observations [59]. Second, the effect of selenite and MSeA on the fractional ^13^C enrichment of the PC-derived uracil ring of UTP (■ Figure 4A,B(c)) or cytosine ring of CTP (■ Figure 4A,B(d)) in A549 cells mirrored that of the precursor Asp (■ Figure 4A,B(b)) for both tracer studies. The same case applied to the ^13^C levels (■ Figure 4A,B(e) for Asp; Figure 4A,B(f) for the uracil unit of UXP). Also noted was the reduced ^13^C enrichment of PDH-derived Asp (■ Figure 4A,B(b)) and uracil/cytosine rings (■ Figure 4A,B(c,d)) elicited by MSeA or selenite treatment. These data suggest a direct block of PC by MSeA or PC/GLS suppression by selenite in A549 cells, which led to an attenuated Asp synthesis and in turn reduced de novo synthesis of pyrimidine nucleotides. Such a block in the anaplerotic inputs into the Krebs cycle could also depress the canonical (PDH-initiated) Krebs cycle activity as we have previously observed with the effect of the PC knockdown (KD) in A549 cells [61]. MSeA-induced inhibition of UTP or CTP synthesis from Glc or Gln in H1299 cells may also be mediated via a direct block of PC.

However, SeM did not significantly alter Asp synthesis via PC-initiated Krebs cycle activity (■ Figure 4A,B(e)) but enhanced that via the canonical Krebs cycle activity (■ Figure 4A,B(e)) in both A549 and H1299 cells. These data contrasted with the SeM-induced decrease in the fractional enrichment of ^13^C-uracil-UTP/-cytosine-CTP (Figure 4A(c,d)) but not in the level of ^13^C-uracil-UTP (Figure 4A(f)) in ^13^C_6_-Glc traced A549 and H1299 cells. This points to reliance on non-Glc source(s) (e.g., Gln) of Asp for sustaining pyrimidine synthesis, which is consistent with enhanced Asp synthesis from Gln (Figure 4B(b,e)) to maintain ^13^C-uracil-UTP levels (Figure 4B(f)) in SeM-treated cells. In contrast, selenite depleted Gln-derived Asp (Figure 4B(b,e)) to a higher extent than that in ^13^C-uracil-UTP (Figure 4B(c)) or ^13^C-cytosine-CTP (Figure 4B(d)) in H1299 cells. This could be due to the selenite-induced GAC suppression (Figure S5B(f)) which blocks Asp synthesis, but also the lack of selenite’s effect on the expression of the key pyrimidine synthesis enzyme carbamoyl phosphate synthetase 2, aspartate transcarbamylase, and dihydroorotase (CAD) (Figure S5B(h)) which continue pyrimidine synthesis in H1299 cells. In contrast, suppressed expression of CAD by selenite and MSeA in A549 cells would aggravate the block of pyrimidine synthesis due to reduced Asp availability. Reduced CAD activity would also contribute to the attenuated GLS-independent glutaminolysis described above.

Taken together, our data suggest that inhibition of PC by MSeA or suppressed expression of GLS isoforms, PC, and CAD by selenite plus compromised ETC activity and thus DHODH action may underlie reduced pyrimidine synthesis in A549 cells. This is also consistent with the association of marginally attenuated pyrimidine synthesis with much fewer expression changes in these enzymes in SeM-treated A549 cells or selenite-treated H1299 cells. The differential capacities of MSeA, selenite, and SeM in blocking pyrimidine synthesis further reflected their effect on cell proliferation (Figure S1).

### 3.9. MseA and Selenite Block Purine Ring Synthesis in A549 Cells via Reduced Gly and Ribose Synthesis

FT-ICR-MS analysis of nucleotides also revealed a reduced level and/or fractional enrichment of ^13^C-adenine-ATP (d,h) and ^13^C-quanine-GTP € by MSeA and selenite but much less so by SeM in ^13^C_6_-Glc-traced A549 cells (Figure S7A). Purine synthesis in H1299 cells was also attenuated by MSeA but less so by selenite or SeM. Likewise, ^13^C incorporation from ^13^C_6_-Glc into the ribose subunit of ATP or GTP followed a similar trend as that into the base unit in A549 or H1299 cells (Figure S7A(d,e,h)). The response of the ^13^C enrichment patterns of adenine and guanine bases to the three Se agents in both cells correlated largely with those of the precursors ^13^C-Ser (Figure S7A(b,f)) and -Gly (Figure S7A(c,g)). This suggests that the inhibitory effect of the three Se agents on purine biosynthesis is at least in part mediated via the block of Ser/Gly synthesis, which occurs from ^13^C_6_-Glc via glycolysis and the 3-phosphoglycerate-Ser-Gly pathway (Figure S7A(a)) [57]. This is consistent with the suppression of two key enzymes, 3-phosphoglycerate dehydrogenase (PHGDH) and phosphoserine aminotransferase 1 (PSAT1), by selenite and MSeA in A549 cells but not by selenite and SeM, and less significantly by MSeA, in H1299 cells (Figure S5B(i,j)). We also noted a buildup of ^13^C_3_-Ser and total ^13^C-Ser levels in selenite-treated A549 cells (Figure S7A(f)) relative to control cells, which could result from attenuated conversion of Ser to Gly via the serine hydroxymethyl transferase (SHMT) activity. Consistent with this was the suppression of both mitochondrial (SHMT2) and cytoplasmic (SHMT1) isozymes by selenite in A549 but not in H1299 cells (Figure S5B(k,l)). MSeA and SeM variably suppressed SHMT1 but not SHMT2 expression in both cell types. Similar to the response pattern of SHMT1 was that of the mitochondrial glycine decarboxylase (GLDC) (Figure S5B(m)), which converts Gly to one-carbon precursors for purine synthesis. The differential suppression of these Ser synthesis and one-carbon pathway enzymes presumably mediated the distinct effect of selenite on purine nucleotide biosynthesis in A549 versus H1299 cells.

Furthermore, reduced purine synthesis was evidenced by the decreased extent of ^15^N incorporation from ^13^C_5,_^15^N_2_-Gln into the adenine ring of ATP and guanine ring of GTP with the efficacy of selenite > MSeA ≥ SeM in both cell lines (Figure S7B(b)), which tracked the effect of selenite and MSeA observed by *in-cell* glutaminolysis assays (Figure 3B). It is plausible that reduced purine synthesis also contributes to the attenuated glutaminolytic activity by MSeA and selenite. Altogether, the differential block in Ser biosynthesis and one-carbon metabolism could account for the variable effect of the three Se compounds on the synthesis of purine nucleotides and in turn on the proliferation of A549 and H1299 cells.

### 3.10. MSeA, Selenite, and SeM Differentially Block Glucose or Gln-Fueled Fatty Acyl Synthesis but Not Glycerol Backbone Incorporation into Lipids

**Figure 5:**
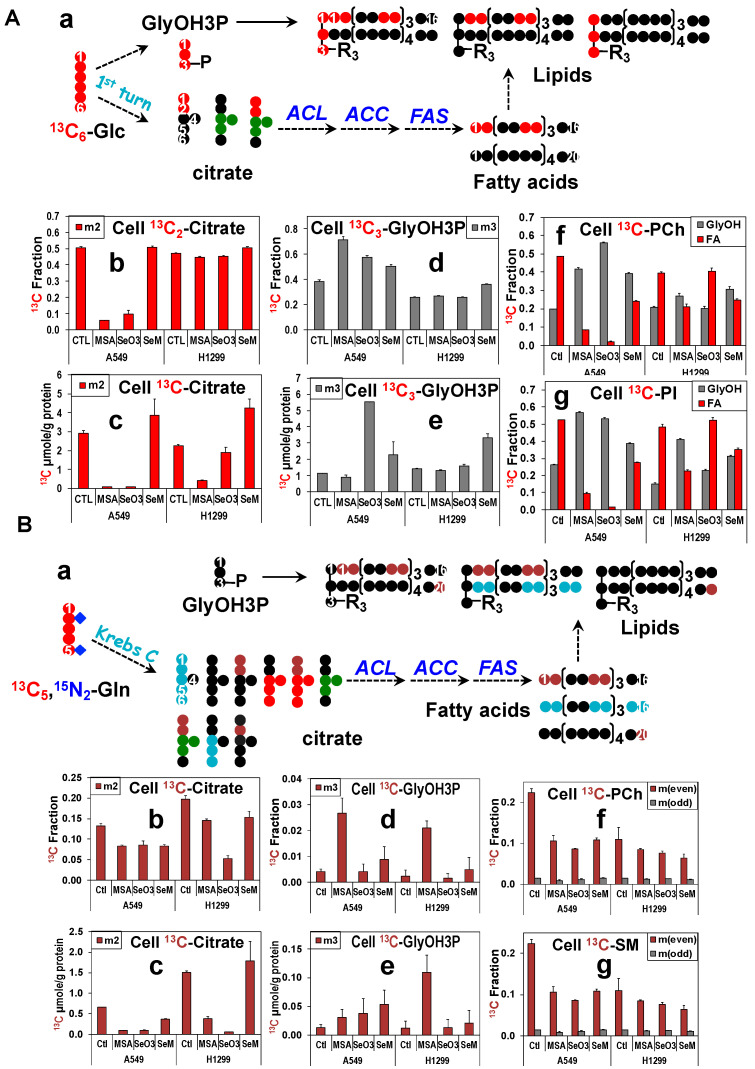
The distinct capacity of MSeA, selenite, and SeM in blocking fatty acyl synthesis from glucose or Gln in A549 and H1299 cells. A549 or H1299 cells were treated with 5 µM MSeA, 6.25 µM selenite, or 500 µM SeM for 24 h with ^13^C_6_-Glc (n = 3) (**A**) or ^13^C_5_,^15^N_2_-Gln (n = 2) (**B**) as tracer, as described in Methods. Polar metabolites (**A**(**b**–**e**),**B**(**b**–**e**)) of cells were analyzed by GC-MS while lipids (**A**(**f**,**g**),**B**(**f**,**g**)) were measured by FT-MS. Lipid synthesis from ^13^C_6_-Glc and ^13^C_5_,^15^N_2_-Gln was traced in (**A**(**a**)) and (**B**(**a**)), respectively. ●, ● in (**A**(**a**)) ^12^C, ^13^C from glycolysis + Krebs cycle ●, ●, ●/●/● via PDH and PC, ◆ in (**B**(**a**)) ^12^C, ^13^C from glutaminolysis + Krebs cycle, ^13^C from glutaminolysis + Krebs cycle + ME + PC, ^15^N, respectively. ❚: ^13^C fraction derived from the ME pathway in Figure 3. mx: mass isotopologues of metabolites with x the number of ^13^C atoms. GlyOH3P: glycerol-3-phosphate; PCh: phosphatidylcholines; SM: sphingomyelins; ACL: ATP-citrate lyase; ACC: acetyl CoA carboxylase; FAS; fatty acid synthase. See Table S12 for statistical analysis.

As shown in Figure 5, another product of the Krebs cycle, citrate, is the requisite precursor to fatty acid biosynthesis from glucose (cf. scheme Figure 5A(a)) or Gln (Figure 5B(a)) while glucose-derived glycerol-3-phosphate (GlyOH3P) supplies the glycerol backbone (GlyOH) of lipids. It should be noted that only carbons at the C1,2 position of citrate are incorporated into fatty acids, which means that PC-derived ^13^C-citrate (●, Figure 5A,B(a)) does not directly support fatty acid synthesis. MSeA and selenite attenuated the level (Figure 5A(c)) and fractional enrichment (Figure 5A(b)) of ^13^C_2_-citrate derived from ^13^C_6_-Glc in A549 cells, which was accompanied by reduced extent of ^13^C incorporation into the fatty acyl chains (❚ FA) of two abundant classes of phospholipids (PLs), phosphatidylcholines (PCh, Figure 5A(f)), and phosphatidylinositols (PI, Figure 5A(g)). SeM also reduced the extent of ^13^C incorporation into FA in A549 cells but had no effect on the synthesis of ^13^C_2_-citrate. These data suggest that reduced Krebs cycle activity plays a key role in attenuated lipid synthesis in MSeA- and selenite-, but not in SeM-treated A549 cells. In contrast, the other precursor ^13^C_3_-GlyOH3P was enhanced in fractional enrichment (Figure 5A(d)) and/or level (Figure 5A(e)) by all three Se agents, which resulted in the increased enrichment in the ^13^C_3_-GlyOH backbone (❚) of PCh and PI. This enhancement could reflect a compensatory increase in the salvage of existing (unlabeled) fatty acids for lipid production.

These effects of the three Se agents on Glc-fueled lipid synthesis were similarly observed for Gln-fueled lipid synthesis in A549 cells (Figure 5B(c–g) except that the treatment responses for MSeA/selenite and the Gln contribution to lipid synthesis were largely less in extent (e.g., Figure 5B(b–f) versus Figure 5B(a–f) ). The low level of ^13^C_3_-GlyOH3P produced from ^13^C_5_,^15^N_2_-Gln via gluconeogenesis could contribute to the lipid species bearing three and the odd number of ^13^C (❚ m(odd) or ^13^C_odd_, Figure 5B(b–f,g). However, we noted a deviation in the response patterns of the fractional enrichment in ^13^C_odd_-lipids (❚ Figure 5B(b–f,g)) from that of ^13^C_3_-GlyOH3P (Figure 5B(b–d,e)), e.g., no MSeA-induced large increase in the enrichment of ^13^C_odd_-lipids to that observed for ^13^C_3_-GlyOH3P. Based on the pathway schemes in Figure 3, ^13^C_1_-citrate (●) can be produced via the mitochondrial ME2 exchange reaction, which can lead to the synthesis of FA and lipids with odd numbers of ^13^C (^13^C_odd_) (Figure 5B(a)), thereby confounding the labeling patterns of ^13^C_odd_-lipids. Lipids with even numbers of ^13^C (❚ m(even) or ^13^C_even_, (Figure 5) can be produced via ^13^C_2_-citrate synthesis from the RedC-ME1 and/or the PDH-ME2 pathways (Figure 3), which was the dominant route of FA synthesis as we have reported for P493 cells [63]. We saw treatment-induced attenuation in the fractional enrichment of the ^13^C_even_-lipids in A549 cells (Figure 5B(b–f,g), which reflected the changes in ^13^C_2_-citrate (Figure 5B(b,c). Correspondingly, we saw reduced levels of ME1 (Figure S5B(n)) and ME2 (Figure S5B(o)) in selenite and SeM-treated A549 cells, which is expected to contribute to the attenuated Gln-fueled lipid synthesis. However, MSeA had no effect on the ME1 or ME2 expression, which suggests a different mechanism for its inhibition of lipid biosynthesis. We then probed the response of ATP-citrate lyase (ACLY) and fatty acid synthase (FASN) (two key enzymes in fatty acid synthesis) to treatments and found their suppression by MSeA, which may well contribute to MSeA inhibition of both Gln- and Glc-fueled lipid synthesis. Likewise, suppressed ACLY and FASN expression by selenite and SeM would attenuate lipid synthesis from both Gln and Glc in A549 cells (Figure S5B(p,q)).

For H1299 cells, only the MSeA treatment elicited a consistently reduced synthesis of ^13^C_2_-citrate (Figure 5B(a–c)) and ^13^C-FA-lipids (Figure 5B(a–f,g)) from the Glc tracer (Figure 5), which point to a key role of the attenuated Krebs cycle in reducing lipid synthesis. Regardless of the changes in ^13^C_2_-citrate derived from the Glc (Figure 5B(a–c)) or Gln tracer (Figure 5B(b,c)), selenite did not significantly alter the ^13^C labeling of lipids (Figure 5(A(f,g),B(f,g)). This may be related to the enhanced expression of ACLY and FASN in selenite-treated H1299 cells. SeM reduced ^13^C labeling of lipids but did not block ^13^C_2_-citrate synthesis from ^13^C_6_-Glc. The former can be attributed to the suppressed ACLY expression in H1299 cells (Figure S5B(p)). Altogether, our data indicate that (1) Glc, and to a lesser extent Gln, fuel lipid synthesis in A549 and H1299 cells; (2) MSeA and selenite block lipid synthesis via inhibition of the canonical Krebs cycle activity, as well as suppression of ACLY and FASN, while suppression of ME1 and ME2 adds to the attenuation of lipid synthesis by selenite and SeM in A549 cells; (3) MSeA and SeM inhibition of lipid synthesis occurs in both A549 and H1299 cells, but selenite blocks lipid synthesis only in A549 cells; (4) the change in patterns of lipid synthesis in response to the three Se agents are consistent with their effects on cell proliferation.

### 3.11. PC or GLS1 Knockdown Inhibits Lung Cancer Cell Proliferation but Their Effects on ROS Production, Cell Cycle Arrest, and Cell Death Are Cell Type-Dependent

The above SIRM data suggest that blocking PC and/or GLS1 functions played a significant role in MSeA and selenite’s ability to inhibit lung cancer cell proliferation. We have recently shown GLS1 suppression to partly account for selenite toxicity in A549 cells [91]. We have also shown that *PC* KD in A549 and H1299 cells inhibited cell proliferation [61]. However, it is unclear how H1299 cells respond to *GLS1* suppression. To address this, we transformed the two cell lines with lentivirus-based shRNA vectors containing none (shEV), scrambled sequence (shScr), or two sequences each against *PC* (shPC54,55) or *GLS1* (shGls35,36) as described in the Supplementary Materials. Expression of each target protein was eliminated by the two vector constructs in both cell lines (Figure S8A–D(a)). PC or GLS1 suppression was accompanied by large decrease in proliferation of both cell lines (Figure S8A–D(b)). This result suggests that failure to suppress PC and GLS1 expression (Figure S5B) underlies the much lower toxicity of selenite in H1299 than in A549 cells (Figure S1B,C). Also noted was that *GLS1* KD enhanced PC expression in both cell lines (Figure S8C–D(b)), as we have observed previously for A549 cells [63]. This could be a compensatory response to help sustain cell growth when Gln-based anaplerosis is blocked [91].

We also examined if *PC* or *GLS1* KD induced ROS production, cell cycle arrest, and cell death for comparison with the MSeA or selenite effect on A549 and H1299 cells. Figure S8A showed that shPC55 elicited > 10-fold increase in ROS production (c), cell cycle arrest at G2/M phase (d), and more apoptotic than necrotic cell death (c) in A549 cells while shPC54 was less effective in inducing ROS production and cell death with cell cycle arrested at the G1 phase. These effects of shPC55 were akin to those of selenite in A549 cells. However, PC suppression altered ROS production and cell cycle distinctly from the inhibition of PC enzyme activity by MSeA (Figure S2 versus Figure S8). Although selenite suppressed GLS1 expression in A549 cells, the effect of *GLS1* KD with two different vectors did not entirely agree with those elicited by selenite. Like selenite, shGLS35 highly enhanced ROS production (c) and induced apoptotic cell death (e) but arrested cell cycle at the G1 phase (d) (Figure S8), which differed from the G2/M arrest by selenite (Figure S2C). Relatively, shGLS36 induced less ROS production and apoptotic cell death but arrested cell cycle at both G1 and G2/M phases (Figure S8C(d)); the latter was akin to the selenite effect on cell cycle. Off target effect(s) may account for the distinct action of the two sh vectors for PC and GLS1. Together, these results suggest that PC suppression may underlie selenite-induced ROS production, cell cycle arrest, and cell death in A549 cells. They also revealed the divergent consequence of PC suppression (induced by selenite) versus PC inhibition (induced by MSeA) in disrupting these cellular processes. Such divergence indicates that compromised PC activity alone cannot account for selenite’s effect on these processes. In addition, it is possible that the PC-promoting effect of GLS1 suppression versus PC-suppressing effect of selenite could contribute to the divergent behavior between *GLS1*-KD and selenite-treated A549 cells.

For H1299 cells, again PC KD with shPC55 enhanced ROS production (c) arrested cell cycle at the G2/M phase (d) and a higher level of apoptosis than necrosis (e) (Figure S8B), which is similar to the response of A549 cells to PC KD (Figure S8A), albeit at a reduced magnitude. shPC54 also elicited a similar response in these processes except for a higher ROS production in shPC54 than in shPC55-treated cells, opposite to the case for A549 cells. This again could be attributed to off target effect(s). *GLS1* KD by both shGLS35 and shGLS36 also induced ROS production (c), cell cycle arrest at G2/M phase (d), and a higher level of apoptosis than necrosis (e) in H1299 cells (Figure S8D). These responses were not evident in selenite-treated H1299 cells (Figure S2), which is to be expected as selenite did not efficiently suppress PC (d), nor GLS1 (e) (Figure S5B).

In essence, failure to suppress *GLS1* and *PC* expression may underlie the lower growth effects of selenite in p53-null H1299 than in wildtype p53 A549 cells while the different mode of PC inactivation (suppression versus inhibition) and differential suppression of GLS1 expression can contribute to the distinct action of selenite versus MSeA in lung cancer cells.

### 3.12. MSeA or Selenite Block Pyruvate Carboxylation and/or Glutaminolysis, Which Was Accompanied by Necrosis in Ex Vivo Organotypic Cultures of Human NSCLC Tissues

**Figure 6:**
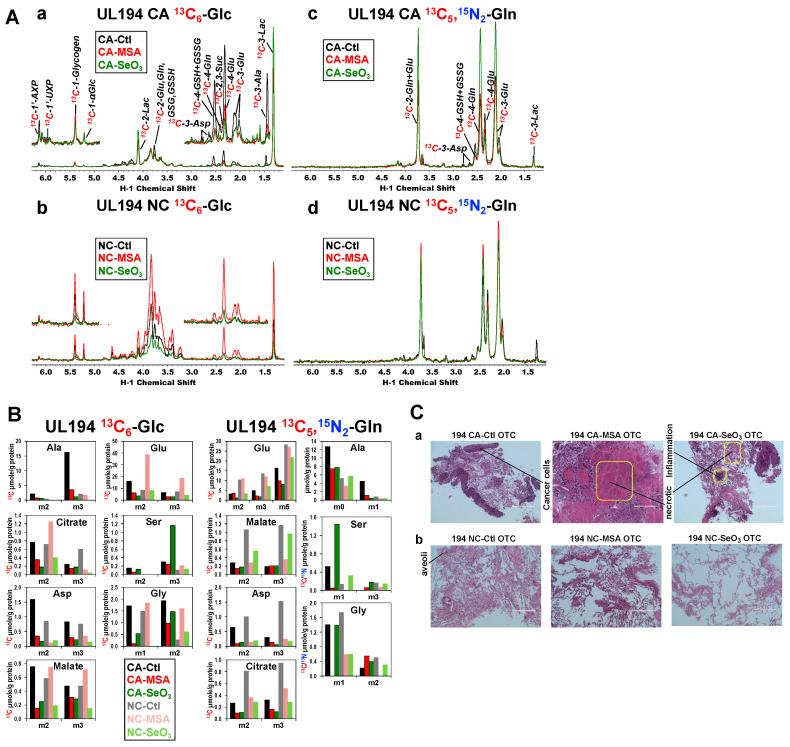
MSeA and selenite induce similar metabolic perturbations in ex vivo CA lung OTC of an NSCLC patient as in NSCLC A549 cells. The matched pair of CA and NC lung OTCs of UL194 patient were treated with 10 µM MSeA or 6.25 µM selenite for 24 h with ^13^C_6_-Glc or ^13^C_5_,^15^N_2_-Gln as tracer, as described in Methods. Polar metabolites of tissue extracts were analyzed by 1D HSQC NMR (**A**) and GC-MS (**B**) while a subsample of tissues was stained with H&E (**C**). ❚, ❚, ❚: Ctl, MSeA, and selenite-treated CA OTC (**a**); ❚, ❚, ❚: Ctl, MSeA, and selenite-treated NC OTC (**b**), respectively. m0–3: mass isotopologues of metabolites with 0–3 ^13^C atoms. N = 1; bar represents 400 µm.

We then asked if the metabolic perturbations induced by MSeA, selenite, and SeM in human lung cancer cells translate into human lung cancer tissues. We have previously established organotypic cultures (OTC) of thinly sliced cancerous (CA) and matched non-cancerous (NC) lung tissues freshly resected from NSCLC patients and have shown that the metabolic reprogramming in ex vivo CA lung OTC recapitulate that *in-patient* [80,90,91,99,100]. Here, we tracked the fate of ^13^C_6_-Glc and/or ^13^C_5_,^15^N_2_-Gln in matched pairs of CA and NC OTC from eight NSCLC patients. Depending on of the size of the resected tissue available, the OTC were treated with none, 10 µM MSeA, 6.25 µM selenite, and/or 500 µM SeM for 24 h. Figure 6 showed the response of CA and NC OTC from patient UL194 to MSeA or selenite in terms of the labeling patterns of central metabolites derived from ^13^C_6_-Glc or ^13^C_5_,^15^N_2_-Gln analyzed by 1D HSQC (Figure 6A) and GC-MS (Figure 6B). With ^13^C_6_-Glc as tracer, we saw enhanced ^13^C labeling in lactate but reduced ^13^C incorporation into Ala, the Krebs cycle metabolites (succinate or Suc, Glu, Asp), GSH + GSSG, and the ribose unit of adenine nucleotides (AXP) in both MSeA (**–**) and selenite-treated (**–**) versus control (**–**) CA OTC (Figure 6A(a)). There were no such responses to selenite but stimulatory responses to MSeA in the matched NC OTC (Figure 6A(b)). With ^13^C_5_,^15^N_2_-Gln as tracer, selenite induced a buildup of ^13^C-Gln and depletion of the glutaminolytic products including ^13^C-Glu, -GSH + GSSG, and -Asp in CA OTC (Figure 6A(c)). A similar depletion of these ^13^C labeled products was elicited by MSeA in CA OTC without the buildup of ^13^C-Gln. These effects of selenite and MSeA on CA OTC recapitulated those on A549 cells in Figure 2 and Figure 3. The effect of selenite or MSeA on the NC counterparts was much attenuated except for the depletion of ^13^C-Asp and -lactate (Figure 6A(d)).

These NMR-observed metabolic changes were consistent with those measured by the GCMS analysis (Figure 6B). In addition, the latter provided evidence for attenuated PC- and PDH-initiated Krebs cycle activity induced by MSeA (❚) and selenite (❚) based on the reduced buildup of ^13^C_3_ and ^13^C_2_ isotopologues of citrate, Asp, and malate, respectively. Moreover, we saw reduced ^13^C_6_-Glc metabolism into Gly and Ser in response to MSeA in CA but not in matched NC OTC. In comparison, selenite reduced ^13^C labeling of Gly in both CA and NC OTC, but this was accompanied by a large buildup of ^13^C_3_-Ser (m3) in CA OTC. The latter was akin to the response of selenite-treated A549 cells (Figure S7A(f)), which can be attributed to SHMT2 suppression (Figure S5B(k)). The buildup of m1 isotopologue of Ser derived from ^13^C_5_,^15^N_2_-Gln in selenite-treated CA OTC is also consistent with SHMT2 suppression. This labeled species is presumably ^15^N_1_-Ser (rather than ^13^C_1_-Ser), the product of phosphoserine aminotransferase 1 (PSAT1) in the Ser synthesis pathway, since its level was much higher than that of the ^13^C labeled species (m3) (Figure 6B). The latter production requires gluconeogenesis (GNG), which was much lower in activity than Ser transamination in lung cancer cells. This is evidenced by the much higher fractional enrichment in the transamination products of ^13^C_5_,^15^N_2_-Gln (e.g., ^15^N-purines, Figure S7B) than the ^13^C labeled GNG products. We further noted a higher buildup of m2-Gly than its precursor m3 Ser induced by MSeA in CA OTC (Figure 6B), which can result from the suppression of mitochondrial GLDC (m), but not SHMT2 (k), as seen in A549 cells (Figure S5). Together, these data suggest that MSeA and selenite block one-carbon metabolism in CA OTC, which may well contribute to their action in reducing ^13^C incorporation from ^13^C_6_-Glc into AXP (Figure 6A(a)). Moreover, the perturbed metabolic changes described above were accompanied by increased necrosis in MSeA-treated CA OTC (a) while no visible damages in the NC counterpart were associated with few metabolic changes (b) (Figure 6C). Since perturbed anaplerotic PC- or GLS-mediated pathways induced by MSeA or selenite led to lung cancer cell death (cf. Figure S2), it is plausible that they also led to tissue damages in UL194′s CA OTC. This is consistent with the absence of these actions and tissue damage in the MSeA-treated NC counterpart.

A similar metabolic action of MSeA (❚) or selenite (❚) on the CA OTC of seven other patients was seen (Figures S9–S11). These included attenuated Krebs cycle and/or glutaminolytic activity, as well as blocked Ser/one-carbon, glutathione and nucleotide metabolism. Also as the case for UL194 (Figure 6A), six out of the seven CA OTCs showed reduced ^13^C incorporation from ^13^C_6_-Glc into glycogen (e.g., Figures S9 and S11). The matched NC OTC responded variably to MSeA or selenite with one or four out of six respectively showing significant inhibition of Glc metabolism (e.g., Figures S10 and S11) while the rest having no or opposite responses (e.g., Figure 6A, Figures S9 and S11A). We also examined the histopathology for the OTC of UL198 patient, which displayed elevated necrosis (30%) and reduced mitotic index (PCNA as marker, Figure S11C(c)) in MSeA or selenite-treated CA OTC versus control CA OTC (0% necrosis). We were unable to subsample a complete set of tissues from the rest of the OTC experiments due to tissue size limitation. For the same reason, we were able to include SeM treatment in only two of the OTC experiments (UL197 and 198). For UL197, SeM at 500 µM (❚) had in general much attenuated or even stimulatory effects (e.g., m2-citrate, m2-lactate) on the ^13^C_6_-Glc metabolism in CA OTC (Figure S10), which is consistent with the effects of SeM on glucose metabolism in lung cancer cells (Figure 2 and Figure S7A). However, glucose metabolism was similarly inhibited in UL198′s CA OTC by SeM as by MSeA or selenite (Figure S11). In contrast, ^13^C_5_,^15^N_2_-Gln catabolism in CA OTC was enhanced by SeM as evidenced by the buildup of ^13^C-Gln, -Glu (Figure S11A(c)), ^13^C-malate, -Asp, -citrate, -Ala, and -Gly (Figure S11B). This anaplerotic response could compensate for the attenuated glucose metabolism to sustain central metabolism, which can in turn relate to the lack of necrosis in SeM-treated CA OTC of UL198, even though the mitotic index was compromised (Figure S11C(c)).

Altogether, the ex vivo CA OTC of eight NSCLC patients were variably sensitive to MSeA or selenite but resistant to a much higher dose of SeM in terms of the central metabolism and tissue damage, which recapitulated the response of lung cancer cells. The NC counterparts were largely resistant to MSeA or SeM with selenite showing compromised Glc metabolism in four out of six NC OTC.

## 4. Conclusions

We showed that lung cancer cells were sensitive to MSeA or selenite at low µM doses but were remarkably resistant to SeM in terms of inhibition of proliferation, apoptosis, and ROS production. ^13^C_6_-glucose and ^13^C_5,_^15^N_2_-Gln-based SIRM studies revealed different metabolic mechanisms that underlie the differential sensitivity of lung cancer cells (A549 cells with WT p53 and p53-null H1299 cells) to the three Se agents. In particular, the ability to block both PC and glutaminase by MSeA and selenite, but not by SeM, correlated well with their ability to inhibit cell proliferation and to induce ROS production. These effects of MSeA and selenite on anaplerosis led to reduced Krebs cycle activity, which in turn attenuated de novo synthesis of nucleotides and lipids, all required for supporting cell proliferation [93,101,102,103,104,105,106,107,108,109]. We have previously shown that PC is activated in vivo in NSCLC patient tumors [61], and that both PC and glutaminolysis are important for supporting lung cancer cell proliferation [91]. We observed an additional cell type-dependent blockade of nucleotide and lipid synthesis by MSeA and selenite, which was related to the suppression of key enzymes in the pyrimidine (CAD)/Ser (PHGDH and PSAT1)-one carbon (SHMT1/2, GLDC) pathways and lipid biosynthetic pathways (ACLY and FASN). These enzymes were also suppressed by SeM in a cell type-dependent manner. The ability of selenite to suppress all the above enzymes plus TFAM/NDUFS1 in A549 but not in H1299 cells could underlie the higher resistance of H1299 to selenite than A549 cells. Pyruvate carboxylation and glutaminolysis were also blocked by MSeA and selenite but not by SeM in ex vivo patient-derived organotypic NSCLC tissue cultures, which correlated with the extent of tissue damage. It should be noted that patient-derived OTC is a unique model for pre-clinical drug evaluation for individual patients due to the maintenance of the integrity of the tumor microenvironment and matched cancer versus non-cancerous tissue design [82]. Although we have shown that selenite and MS blocked anaplerotic PC and glutaminolysis, detailed regulatory mechanisms of these actions will require further studies.

The inefficacy of SeM (even at 500 µM) to block anaplerosis in human lung cancer cells and tissues can be related to the unsuccessful outcome of the SeM-based SELECT and selenized yeast (with SeM as major component)-based chemopreventive trials for NSCLC [35]. In contrast, MSeA and selenite can be more effective anti-cancer and possibly chemopreventive agents at least in part due to their ability to block anaplerosis. Such knowledge will be crucial to the rational design of both therapeutic and chemopreventive strategies in clinical trials.

## Figures and Tables

**Table 1:** Protein Targets and antibodies used for RPPA analyses.

Protein Targets	Vendor	Catalogue Number	Dilution
ACLY	Proteintech Group	15421-1-AP	1:100
CAD	Proteintech Group	16617-1-AP	1:100
CCND1	Proteintech Group	60186-1-Ig	1:100
FASN	Proteintech Group	10624-2-AP	1:100
GAC ^a^	Gift of Dr. S. Dias ^b^		1:3000
GLDC	Proteintech Group	24827-1-AP	1:100
GLS2	Invitrogen	PA5-72963	1:100
KGA/GAC	Proteintech Group	12855-1-AP	1:100
ME1	Proteintech Group	16619-1-AP	1:100
ME2	Proteintech Group	24944-1-AP	1:100
MTATP8	Proteintech Group	26723-1-AP	1:100
NDUFS1	Proteintech Group	12444-1-AP	1:100
PC	Proteintech group	16588-1-AP	1:100
PHGDH	Proteintech Group	14719-1-AP	1:100
PSAT1	Proteintech group	10501-1-AP	1:100
SHMT1	Proteintech Group	14149-1-AP	1:100
SHMT2	Proteintech Group	11099-1-AP	1:100
TFAM	Proteintech Group	19998-1-AP	1:100

^a^ from Western blot performed in 10% polyacrylamide gel with 15 µg protein loaded per sample, which was electrophoresed at 115 V for 3 h, transferred onto nitrocellulose membrane overnight at 10 V in 25 mM Tris/192 mM glycine/0.1% SDS/20% methanol buffer, pH 8.5. The blot was blocked in 5% milk for 1 h, incubated in 1:10,000 diluted anti-rabbit secondary antibody for 1 h, and visualized using the Pierce ECL Western blotting kit (Thermo Scientific). ^b^ see reference [88].

## Data Availability

The datasets used and/or analyzed for the current study are provided in the Figures and Tables, and are available from the corresponding author on reasonable request. The raw data are not currently publicly available because of complexity of deposition of the various data types.

## Supplementary Materials

The following supporting information can be downloaded at: https://www.mdpi.com/article/10.3390/metabo13070774/s1, Figures S1–S11; Tables S1–S12 of statistical analyses. Reference [110] is cited in the Supplementary Materials.
