# Supplementary material for: Differential Inhibition of Anaplerotic Pyruvate Carboxylation and Glutaminolysis-Fueled Anabolism Underlies Distinct Toxicity of Selenium Agents in Human Lung Cancer
Source: Metabolites. 2023 Jun 21;13(7):774. doi: 10.3390/metabo13070774 (PMC10383978; doi:10.3390/metabo13070774)
Supplement: Supplementary file 1 [file metabolites-13-00774-s001.zip › metabolites-2307650-supplementary.pdf]

Figure S1

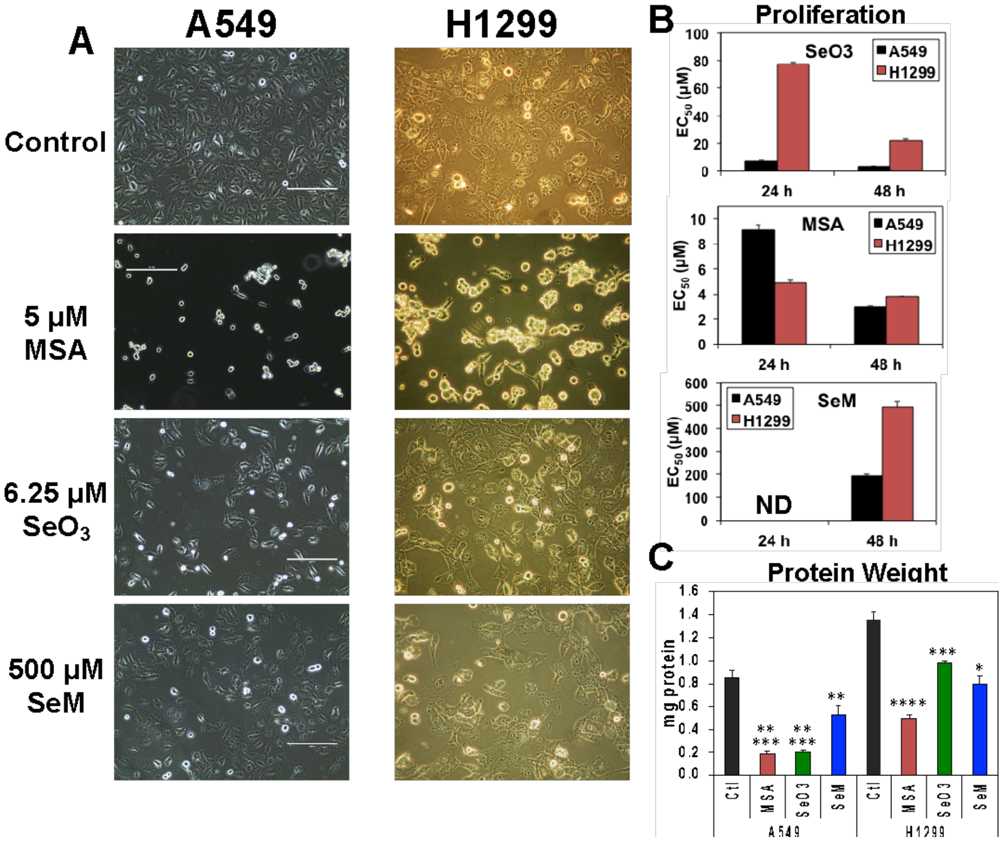

Figure S1. MSA, selenite, and SeM vary in their effects on lung cancer cell morphology and proliferation.

Images in **A** were acquired with 200x magnification (white bar = 200 µm). Doses that attenuated 50% cell population (EC_{50}) were determined in **B**, where the EC_{50} for 24 h of SeM treatment could not be accurately determined (ND) due to the very low toxicity. In **C**, protein weights of A549 and H1299 cells after 24 hr of treatment with control, 5 µM MSA, 6.25 µM selenite, or 500 µM SeM were determined by the BCA method, as described in Supplemental information. *: $q < 0.05$; **: $q < 0.01$; ***: $q < 0.005$; *****: $q < 0.00005$ (n=5).

Figure S2

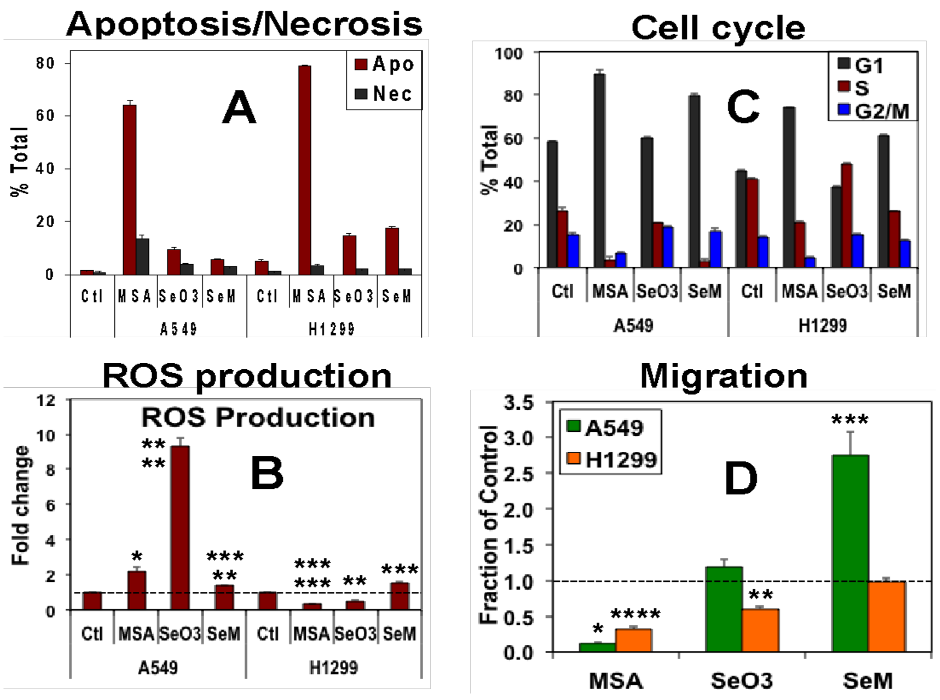

Figure S2. MSA, selenite, and SeM vary in their effects on lung cancer cell death, ROS production, and cell cycle arrest.

A549 and H1299 cells were treated with control, 5 μM MSA, 6.25 μM selenite, or 500 μM SeM for 24 hr (n=3) before flow cytometry analysis of apoptosis/necrosis (**A**), ROS production (**B**), and cell cycle arrest (**C**), as described in Supplemental information. For migration assay (**D**), cells were allowed to migrate for 6 h in the presence of 5 μM MSA, 6.25 μM selenite, or 500 μM SeM. *: $q < 0.05$; **: $q < 0.01$; ***: $q < 0.005$; *****: $p < 0.00005$ (n=5) in **B** and **D**; see **Table S1** for statistical analysis for **A** and **C**.

Figure S3

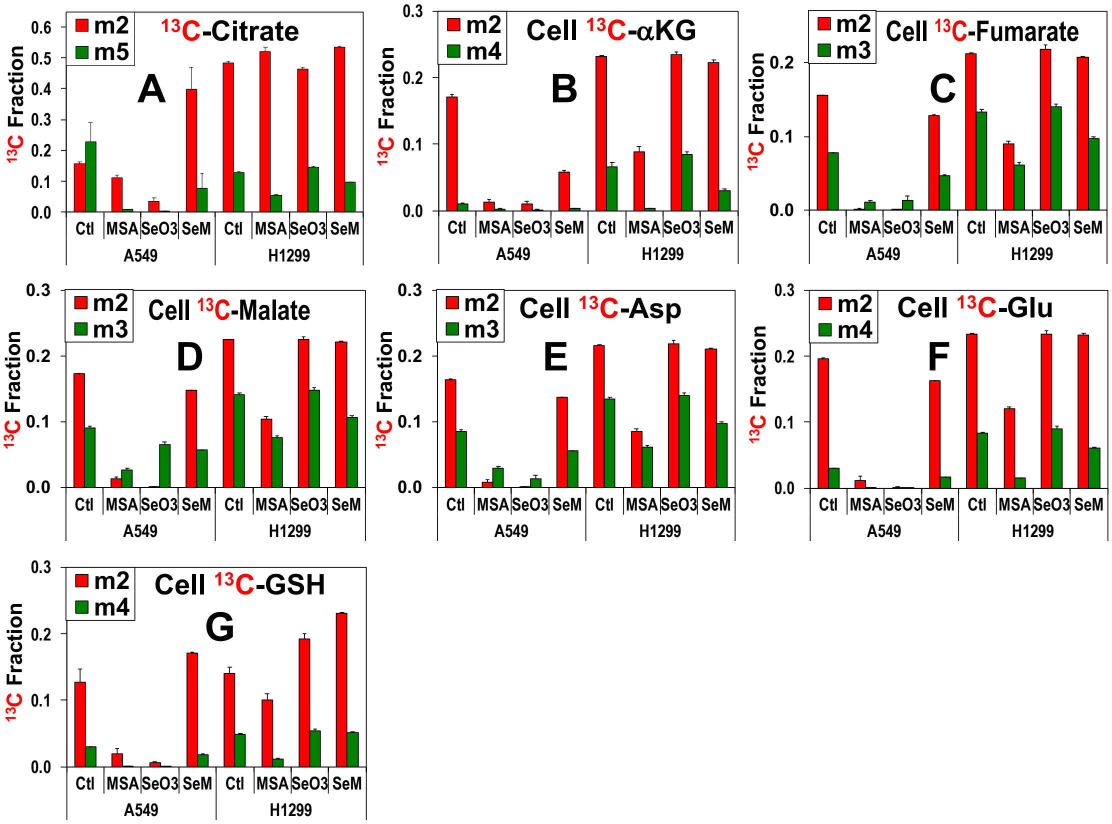

Figure S3. Changes in ^{13}C fractional enrichment of metabolites derived from PC- and PDH-initiated Krebs cycle reactions are cell type- and Se treatment-dependent.

IC-FTMS data from the $^{13}C_6$-glucose tracer experiment in **Fig. 2** were calculated for the fraction of the $^{13}C_2$, $^{13}C_3$, $^{13}C_4$, and $^{13}C_4$ isotopologues (n=2-3). See **Table S4** for statistical analysis.

Figure S4

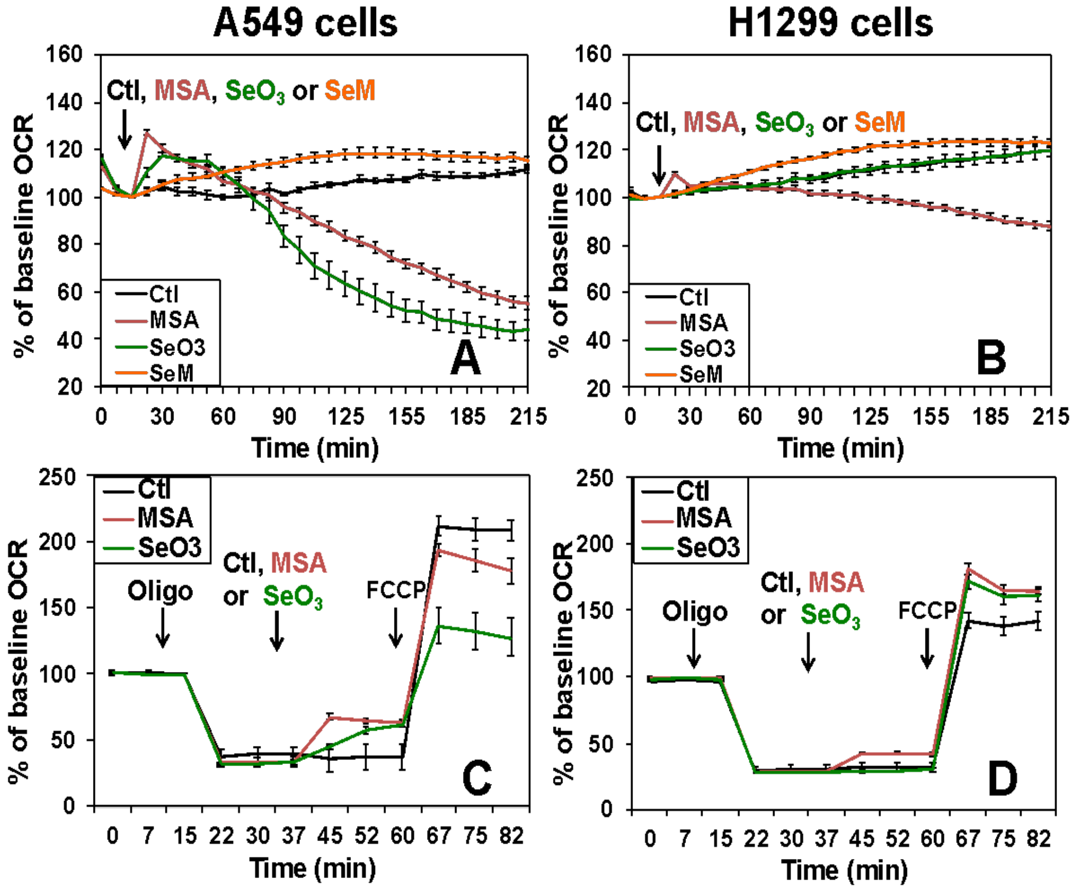

Figure S4. Cell type- and Se treatment-dependent changes in mitochondrial respiration.

Oxygen consumption rate (OCR) was measured in A549 and H1299 cells under control (−), 5 μM MSA (−), 6.25 μM selenite (−), or 500 μM SeM (−) treatment (n=3) using the XF[e]96 analyzer as described in Supplemental information. Se agents, oligomycin (Oligo), or Carbonyl cyanide-*4*-(trifluoromethoxy)phenylhydrazone (FCCP) were added as indicated by arrows.

Figure S5

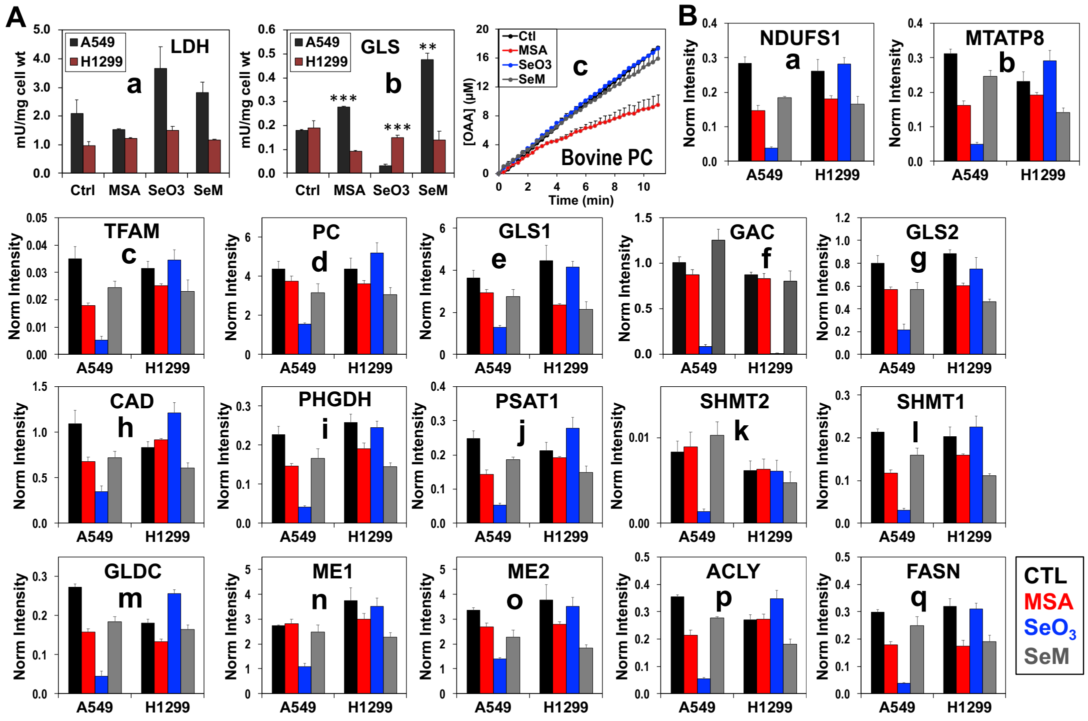

Figure S5. MSA, selenite, and SeM vary in their effects on central metabolic protein expression or activity in A549 and H1299 lung cancer cells.

LDH, GLS, and PC activities in **A** were assayed in crude lysates of A549 and H1299 cells after 24 hr of control, 5 µM MSA, 6.25 µM selenite, or 500 µM SeM treatment. Relative protein quantification in **B** was performed using West blot (**e-f**) or RPPA (the rest). NDUFS1: NADH:ubiquinone oxidoreductase core subunit S1; MTATP8: ATP Synthase F0 Subunit 8; TFAM: mitochondrial transcription factor 1; PC: pyruvate carboxylase; KGA: kidney type glutaminase or glutaminase 1; GAC: glutaminase 1 splice variant; GLS2: glutaminase liver isoform; CAD: carbamoyl synthetase, aspartate transcarbamylase, and dihydroorotase; PHGDH: 3-phosphoglycerate dehydrogenase; PSAT1: phosphoserine aminotransferase 1; SHMT1/2: serine hydroxymethyl transferase 1/2; GLDC: glycine decarboxylase; ME 1/2: malic enzyme 1/2; ACLY: ATP citrate lyase; FASN: fatty acid synthetase; EZH2: enhancer of Zeste homolog 2; CCND1: cyclin D1. **: $q < 0.01$; ***: $q < 0.005$ (n=2); see **Table S9** for the rest of statistical analysis.

Figure S6

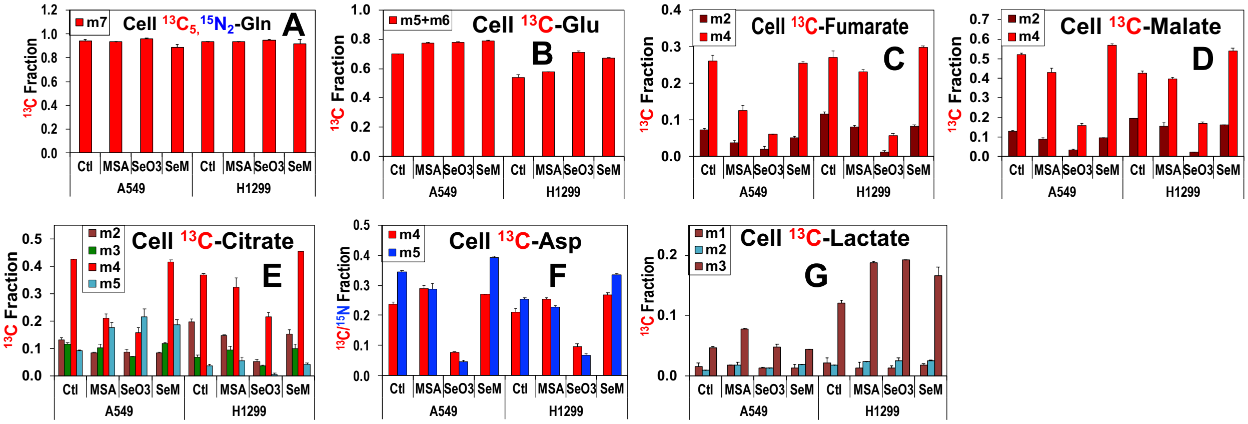

Figure S6. Cell type- and Se treatment-dependent changes in the ^{13}C fractional enrichment of glutaminolytic products.

GC-MS data from **Fig. 3** were calculated for the fraction of the $^{13}C_7$ isotopologue of Gln and the $^{13}C_1$ to $^{13}C_5$ isotopologues of glutaminolytic products (n=2). m4: $^{13}C_4$; m5: $^{13}C_4$,$^{15}N_1$; m5+m6: $^{13}C_5$ + $^{13}C_5$,$^{15}N_1$. See **Table S7** for statistical analysis.

Figure S7

A

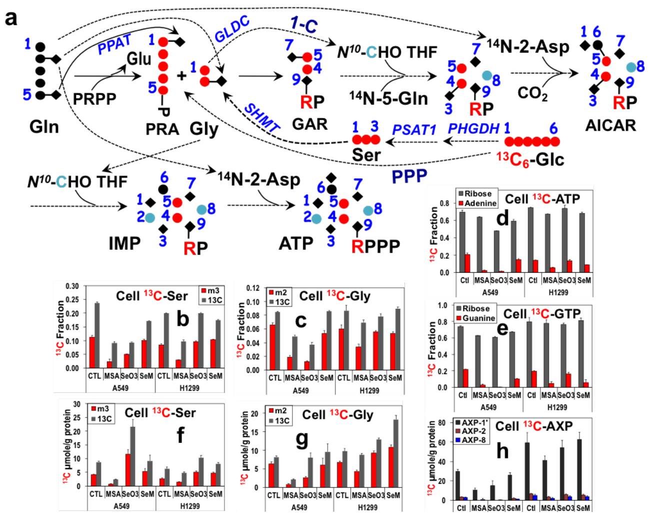

B

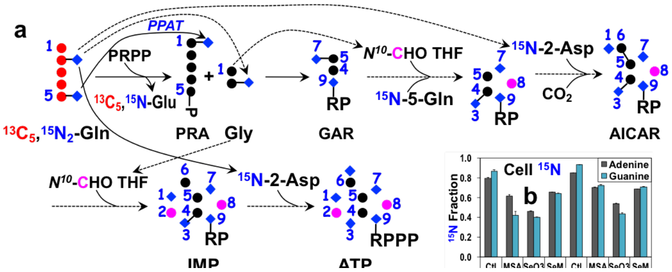

Figure S7. Cell type- and Se treatment-dependent inhibition of purine synthesis.

The precursors of purine ring and the purine nucleotide products were quantified in the same sets of cell extracts as in **Fig. 4**. Atom-resolved tracing from $^{13}C_6$-Glc (**A-a**) or $^{13}C_5$,$^{15}N_2$-Gln (**B-a**) into ATP is shown along with the changes (average of 2 replicates) in the ^{13}C labeling patterns of the Ser/Gly precursor and the products ATP and GTP. **A-b** to **c**/**f** to **g** data were acquired from GC-MS, **A-d** to **e**/**B-b** data from FTMS, and **A-h** data from 1D HSQC NMR analyses. See **Table S5** for statistical analysis in **A** and **B**.

Figure S8

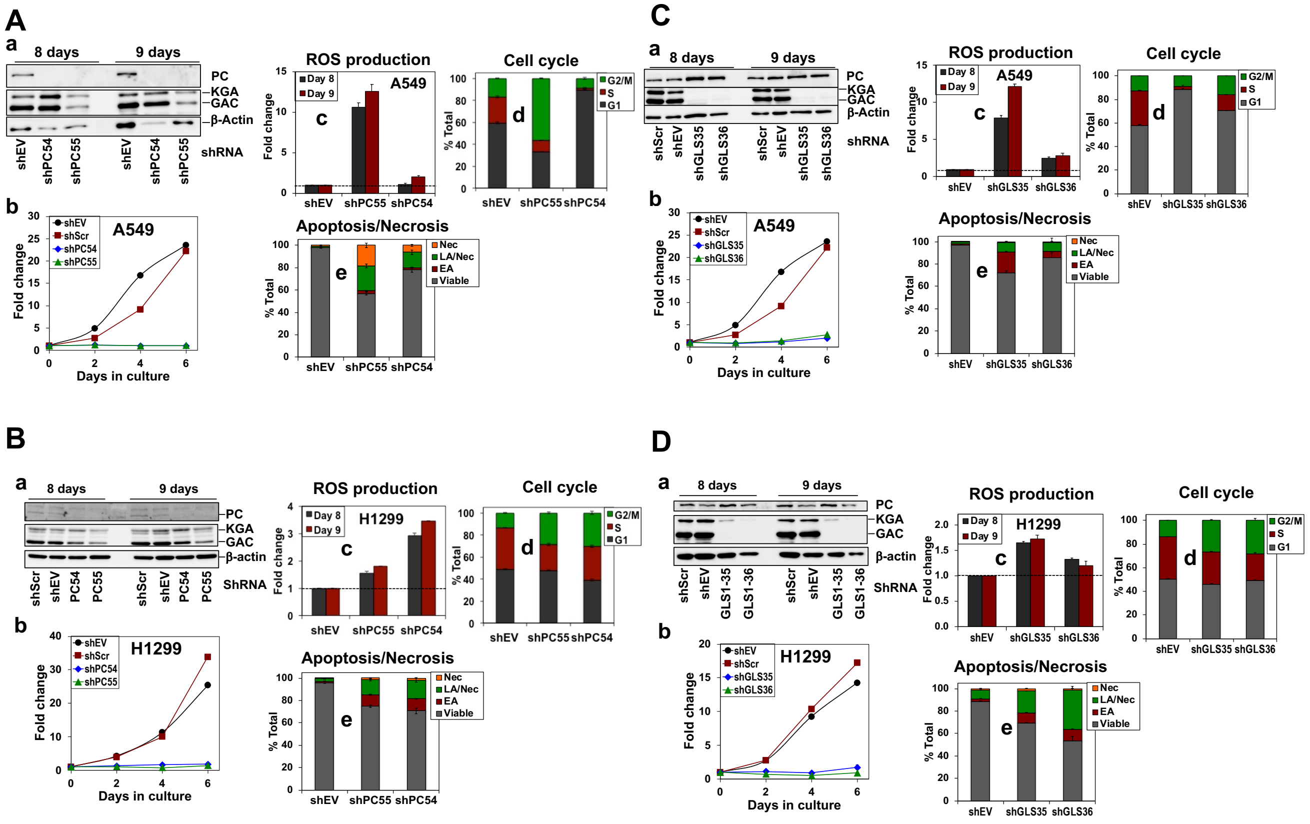

Figure S8. PC/GLS1 knockdown block proliferation but effects on cell death/ROS production/cell cycle arrest are cell type-dependent.

PC or GLS1 (KGA + splice variant GAC) expression in A549 and H1299 cells was attenuated using two each targeted shRNA vectors (shPC54/55 and shGls36/37). Also included were two control vectors each with empty (shEV) or scrambled RNA sequence (shScr), as described in Supplemental information. Changes in PC and GLS1 protein expression in tranduced cells were analyzed by Western blot in **a**. Effects of the knockdown on time-dependent cell proliferation are shown in **b**. Assays for ROS were done 8 and 9 days after shRNA treatments while that for apoptosis/necrosis and cell cycle were done 9 days after shRNA treatment (n=3), as described in Supplemental information. Nec: necrosis; EA/LA: early/late apoptosis. See **Tables S10-11** for statistical analysis.

Figure S9

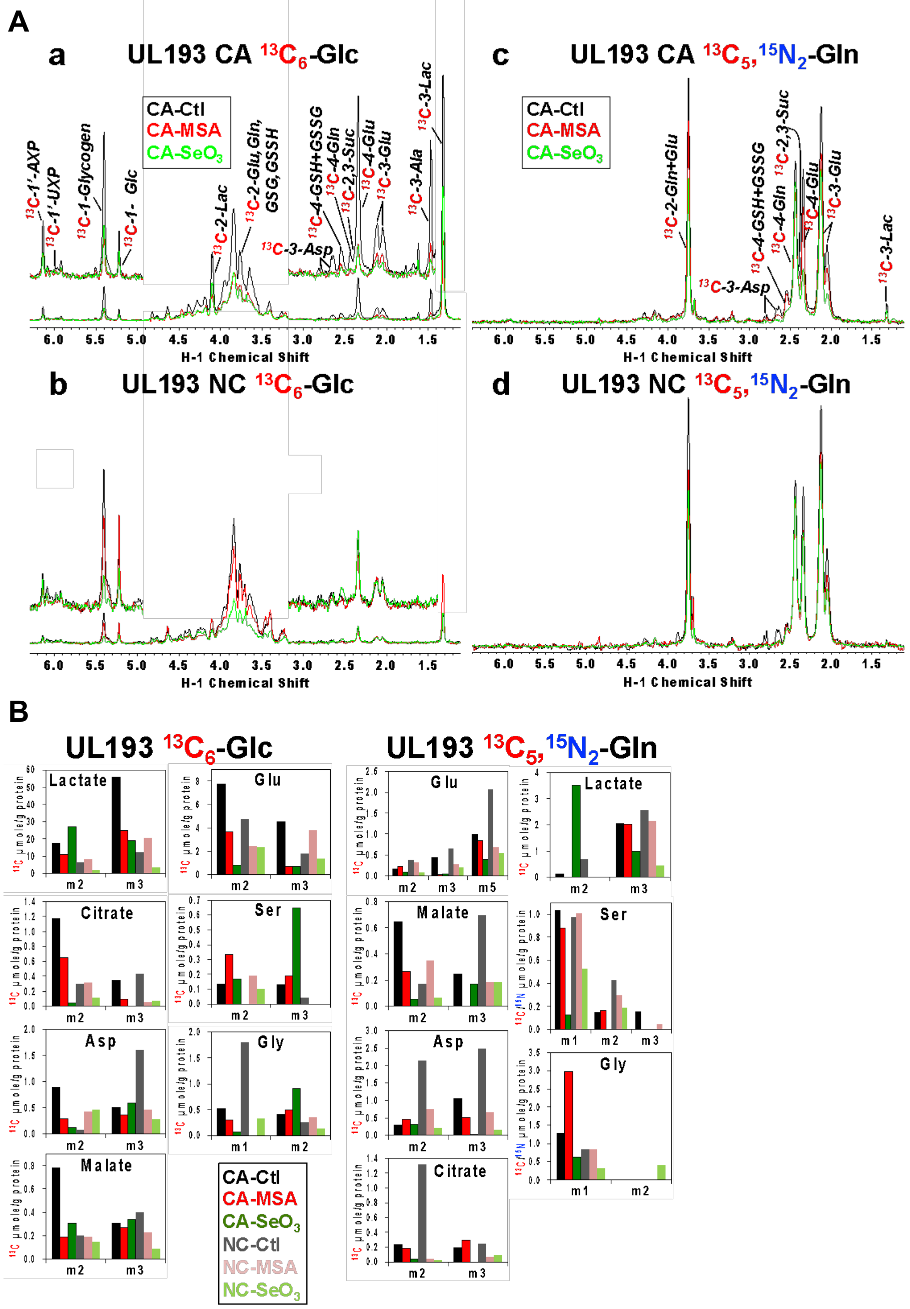

Figure S9. MSA and selenite block PC and glutaminolysis in a second *ex vivo* NSCLC patient-derived organotypic lung cancer tissue culture.

CA and matched NC lung tissues of patient UL193 were thinly sliced and cultured in DMEM medium containing $^{13}C_6$-Glc or $^{13}C_5$,$^{15}N_2$-Gln along with vehicle (Ctl, −), 6.25 μM selenite (−, −), or 10 μM MSA (−, −) for 24 hr as described in Methods. Polar extracts of tissues were analyzed by 1D HSQC NMR (**A**) and GC-MS (**B**) for ^{13}C and/or ^{15}N labeling in Krebs cycle metabolites and amino acids (n=1). The m1 isotopologues of Ser and Gly derived from $^{13}C_5$,$^{15}N_2$-Gln were most likely ^{15}N-Ser and -Gly based on their high abundance in Ctl tissues and the expected prevalence of the transaminase reaction.

Figure S10

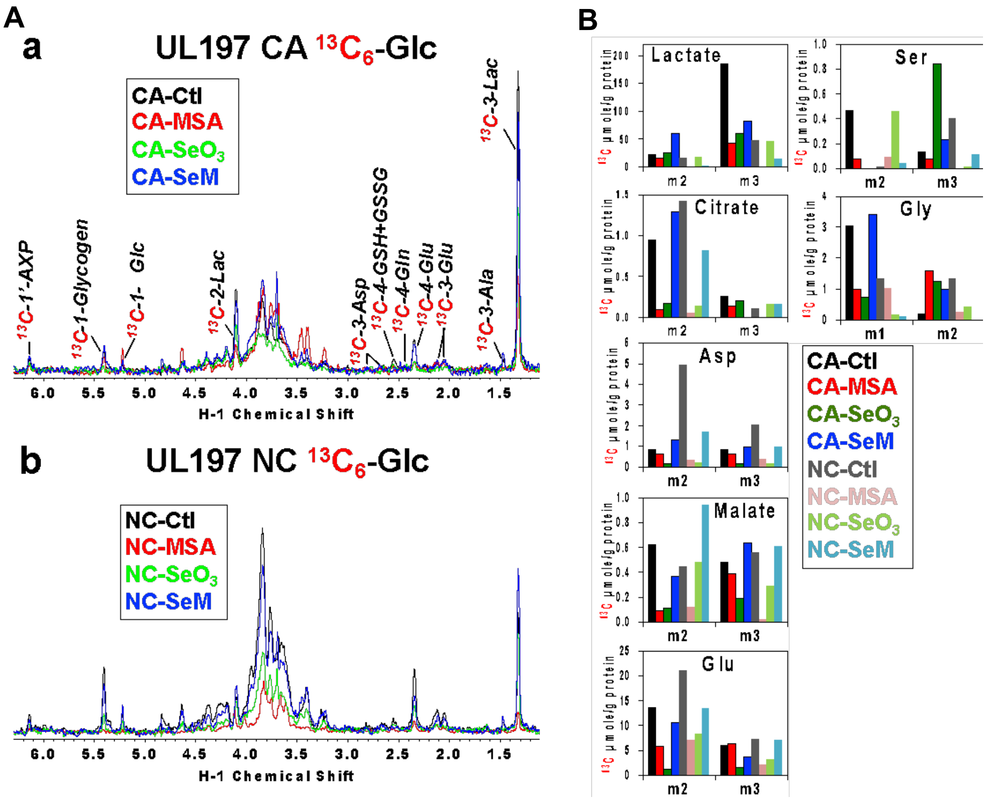

Figure S10. MSA and selenite but not SeM block PC in a third *ex vivo* NSCLC patient-derived organotypic lung cancer tissue culture.

Organotypic tissue culturing and metabolite analysis of NSCLC patient UL197 was performed similarly as in **Figs. 6** and **S9**, except for the use of $^{13}C_6$-glucose only as tracer and the additional 500 μM SeM treatment (−, −).

Figure S11

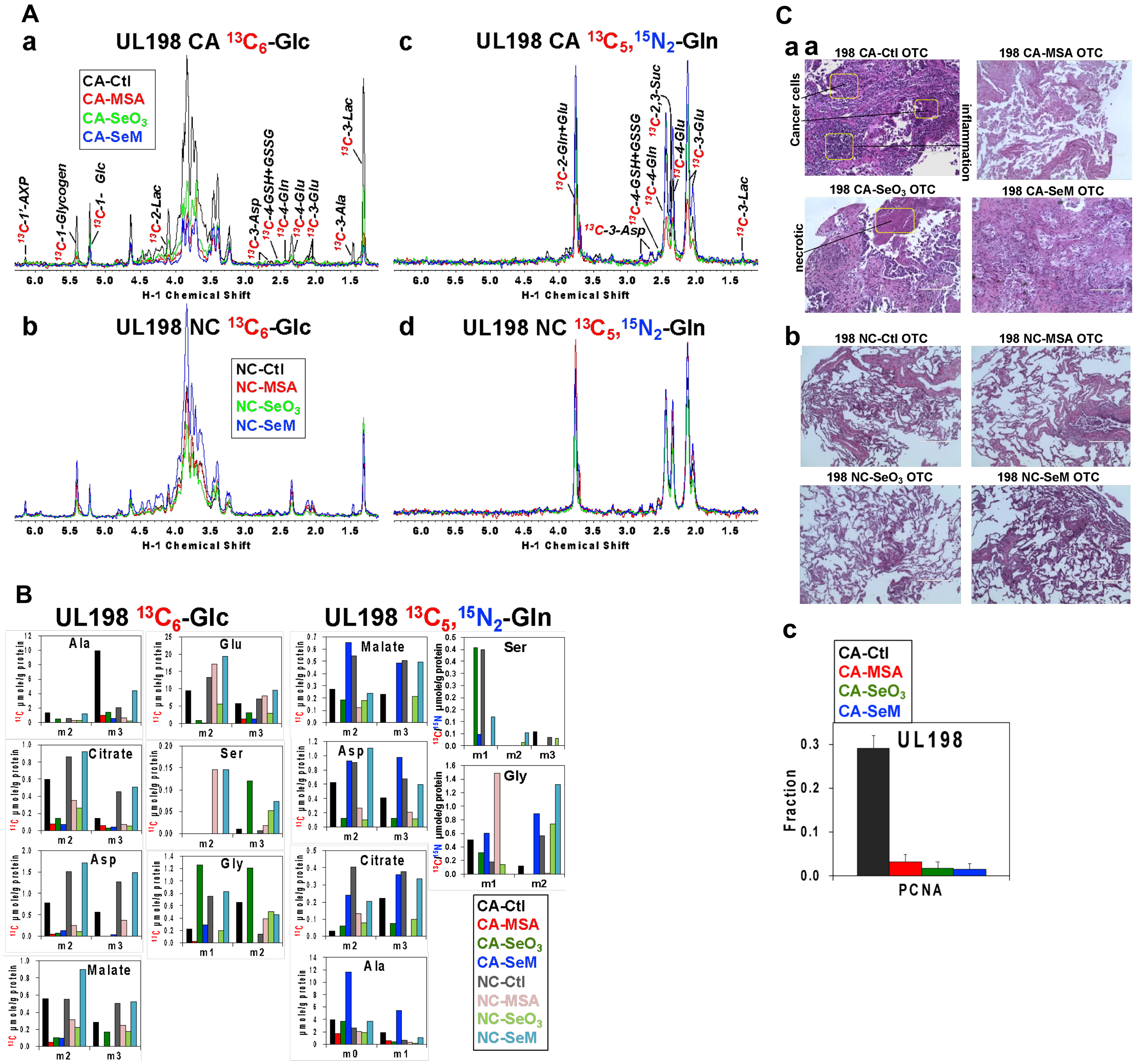

Figure S11. MSA, selenite, and SeM block PC but have variable effects on glutaminolysis in a fourth *ex vivo* NSCLC patient-derived organotypic lung cancer tissue culture.

Organotypic tissue culturing and metabolite analysis of NSCLC patient UL198 was performed similarly as in **Figs. 6** and **S9**, except for the additional 500 μM SeM treatment (−, −).

Table S1. Q values for Se agent-induced changes in cell cycling of A549 and H1299 cells

	**Q for Cell cycle parameters** [a]					
	**A549**			**H1299**		
	Ctl vs MSA	Ctl vs SeO_3	Ctl vs SeM	Ctl vs MSA	Ctl vs SeO_3	Ctl vs SeM
G1	**3.98E-04**	**4.51E-02**	**3.38E-04**	**2.23E-05**	**1.88E-03**	**4.79E-05**
S	**9.10E-04**	**1.06E-02**	**4.13E-04**	**6.30E-05**	**2.51E-03**	**7.39E-05**
G2/M	**5.96E-03**	**4.80E-02**	3.01E-01	**7.93E-05**	1.24E-01	**2.71E-03**
	Q for Apoptosis/Necrosis [b]					
Apo	**1.16E-05**	**1.50E-03**	**1.20E-03**	**2.73E-08**	**1.53E-03**	**8.26E-05**
Nec	**2.76E-03**	**1.25E-03**	**1.72E-03**	**2.30E-04**	**5.51E-03**	**5.96E-03**

[a] Cell cycle analysis was performed as described in **Methods**. The *p*-values were calculated using the two-tailed t-test for % cell in each cell cycle phase for control against those of each of the three Se treatments in **Fig. S2C**. The *p*-values were then corrected for multiple testing using the Benjamin-Hochberg procedure [1] to obtain the Q-values for each parameter. Bolded values denote statistical significance (Q ≤ 0.05).

[b] Apoptosis and necrosis analysis was performed as described in **Methods**. Q-values were calculated as in ref. [110] .

Table S2. **Q values for Se agent-induced changes in NMR-determined concentration of medium metabolites and their ^{13}C labeled isotopologues in $^{13}C_6$-glucose-treated A549 and H1299 cells.**

	Q for concentration [a]					
	**A549**			**H1299**		
	Ctl vs MSA	Ctl vs SeO_3	Ctl vs SeM	Ctl vs MSA	Ctl vs SeO_3	Ctl vs SeM
$^{13}C_3$-Lactate (medium)	**1.48E-03**	**5.77E-03**	**3.42E-02**	**1.32E-02**	1.22E-01	9.97E-02
$^{13}C_6$-Glucose (medium)	1.50E-01	**8.82E-04**	2.41E-01	7.92E-02	1.82E-01	5.16E-02
$^{13}C_3$-Lactate (cell)	**1.30E-03**	**1.95E-03**	**3.91E-03**	6.89E-02	4.91E-01	**2.95E-02**
$^{13}C_3$-Pyruvate (cell)	**1.24E-02**	**1.48E-02**	2.86E-01	8.94E-02	2.44E-01	1.60E-01
$^{12}C_3$-Lactate (cell)	**2.43E-03**	**2.83E-02**	9.75E-02	**4.71E-02**	8.99E-01	**4.77E-02**
$^{12}C_3$-Pyruvate (cell)	**1.41E-02**	**1.48E-02**	8.60E-01	8.17 E-02	1.75E-01	3.64E-01

[a] Medium or cellular metabolite concentrations were determined respectively from ^1H NMR or GC-MS analysis and normalized to soluble protein levels. The *p*-values were calculated using the two-tailed t-test for metabolite concentrations of control against those of each of the three Se treatments in **Fig. 1B**. The *p*-values were then corrected for multiple testing using the Benjamin-Hochberg procedure [110] to obtain the Q-values for each metabolite. Bolded values denote statistical significance (Q ≤ 0.05).

Table S3. **Q values for Se agent-induced changes in concentration of cellular metabolites and their ^{13}C labeled isotopologues in $^{13}C_6$-glucose-treated A549 and H1299 cells.**

	**Q for µmole/g protein [a]**					
	**A549**			**H1299**		
	Ctl vs MSA	Ctl vs SeO_3	Ctl vs SeM	Ctl vs MSA	Ctl vs SeO_3	Ctl vs SeM
$^{13}C_2$-αKG	**1.91E-03**	**7.47E-03**	1.11E-01	3.80E-01	4.85E-01	9.30E-01
$^{13}C_2$-Asp	**2.40E-03**	**3.28E-04**	3.89E-01	**5.88E-04**	9.46E-01	3.00E-01
$^{13}C_2$-Citrate	1.29E-01	**4.37E-02**	5.07E-01	**3.76E-04**	8.81E-01	3.15E-01
$^{13}C_2$-Fumarate	**9.45E-04**	**5.95E-04**	8.63E-01	**3.04E-04**	1.01E+00	8.27E-01
$^{13}C_2$-Malate	**3.62E-04**	**7.94E-05**	6.65E-01	**2.92E-04**	6.76E-01	4.54E-01
$^{13}C_2$-Glu	**2.25E-03**	**2.02E-03**	4.43E-01	**1.08E-03**	5.11E-01	2.57E-01
$^{13}C_2$-GSH	**4.99E-03**	**4.61E-03**	6.92E-01	**4.21E-02**	1.06E+00	7.38E-01
$^{13}C_3$-Asp	**1.49E-02**	**3.71E-03**	5.74E-01	**3.24E-04**	4.20E-01	8.96E-01
$^{13}C_5$-Citrate	**9.12E-03**	**3.80E-03**	1.24E-01	**1.93E-04**	1.01E+00	9.60E-01
$^{13}C_3$-Fumarate	**1.36E-03**	**4.84E-03**	4.02E-01	**6.49E-04**	9.78E-01	6.05E-01
$^{13}C_3$-Mal	**1.44E-03**	**4.70E-03**	5.38E-01	**3.02E-04**	8.92E-01	9.65E-01
$^{13}C_4$-αKG	**1.45E-02**	**1.64E-02**	1.26E-01	**4.04E-02**	**3.88E-02**	5.42E-01
$^{13}C_4$-Glu	**2.49E-03**	**3.21E-03**	6.86E-01	**1.02E-04**	7.87E-01	9.84E-01
$^{13}C_4$-GSH	**8.72E-03**	**8.62E-03**	1.72E-01	**5.56E-04**	9.53E-01	5.19E-02

[a] Metabolite concentrations were determined from IC-UHRMS analysis and normalized to soluble protein levels. The *p*-values were calculated using the two-tailed t-test for metabolite concentrations of control against those of each of the three Se treatments in **Fig. 2**. The *p*-values were then corrected for multiple testing using the Benjamini-Hochberg procedure [110] to obtain the Q-values for each metabolite. Bolded values denote statistical significance ($Q \le 0.05$).

Table S4. **Q values for Se agent-induced changes in fractional distribution of ^{13}C labeled metabolite isotopologues in $^{13}C_6$-glucose-treated A549 and H1299 cells.**

	Q for fractional enrichment [a]					
	A549			H1299		
	Ctl vs MSA	Ctl vs SeO_3	Ctl vs SeM	Ctl vs MSA	Ctl vs SeO_3	Ctl vs SeM
$^{13}C_2$-αKG	**2.65E-05**	**1.95E-04**	**4.14E-04**	**1.24E-04**	6.95E-01	1.23E-01
$^{13}C_2$-Asp	**2.35E-05**	**3.83E-06**	**1.27E-04**	**4.98E-05**	7.31E-01	**4.09E-02**
$^{13}C_2$-Citrate	**4.40E-02**	**1.38E-02**	8.78E-02	**4.69E-02**	1.28E-01	**2.34E-03**
$^{13}C_2$-Fumarate	**1.48E-07**	**1.79E-10**	**1.07E-04**	**2.59E-05**	5.11E-01	8.10E-02
$^{13}C_2$-Malate	**2.66E-05**	**5.32E-06**	**1.99E-04**	**2.01E-05**	9.92E-01	1.00E-01
$^{13}C_2$-Glu	**2.23E-05**	**5.99E-06**	**1.55E-04**	**1.61E-05**	1.02E+00	5.55E-01
$^{13}C_2$-GSH	**9.40E-03**	**2.10E-02**	1.02E-01	**4.35E-02**	1.92E-01	**7.49E-03**
$^{13}C_3$-Asp	**5.49E-04**	**8.02E-04**	**1.41E-04**	**9.88E-05**	1.01E+00	**4.45E-03**
$^{13}C_5$-Citrate	**2.20E-02**	**2.08E-02**	3.69E-01	**1.12E-04**	1.33E-01	**2.21E-03**
$^{13}C_3$-Fumarate	**6.17E-05**	**1.06E-03**	**1.36E-04**	**1.28E-04**	3.19E-01	**3.06E-03**
$^{13}C_3$-Mal	**3.18E-04**	**9.64E-02**	**1.27E-04**	**1.22E-04**	4.75E-01	**3.23E-03**
$^{13}C_4$-αKG	**1.10E-02**	**5.78E-03**	**4.25E-02**	**4.64E-04**	1.12E-01	**3.67E-02**
$^{13}C_4$-Glu	**1.44E-05**	**6.91E-06**	**1.48E-04**	**1.61E-05**	2.73E-01	**2.72E-03**
$^{13}C_4$-GSH	**1.52E-05**	**7.00E-06**	**1.03E-03**	**1.57E-05**	1.15E-01	1.27E-01

[a] The Q values for the fractional distribution of ^{13}C labeled metabolites in **Fig. 2** were calculated similarly as in **Table S3**. Bolded values denote statistical significance ($Q \leq 0.05$).

Table S5. **Q values for Se agent-induced changes in fractional distribution of ^{13}C labeled isotopologues of purine nucleotides and precursors in $^{13}C_6$-glucose-treated A549 and H1299 cells.**

	**Q for ^{13}C fractional enrichment [a]**					
	**A549**			**H1299**		
	Ctl vs MSA	Ctl vs SeO_3	Ctl vs SeM	Ctl vs MSA	Ctl vs SeO_3	Ctl vs SeM
ATP $^{13}C_5$-ribose	7.30E-02	**9.02E-04**	**4.79E-02**	**2.12E-03**	8.90E-01	**1.58E-02**
ATP ^{13}C-ring	**1.02E-03**	5.52E-02	7.49E-02	**6.03E-04**	8.40E-01	**7.55E-04**
GTP $^{13}C_5$-ribose	**3.17E-03**	**7.86E-04**	**3.43E-02**	6.94E-01	1.32E-01	8.59E-01
GTP ^{13}C-ring	**6.01E-04**	**1.64E-05**	**3.83E-03**	**1.55E-02**	8.65E-01	9.17E-02
$^{13}C_3$-Ser	**9.03E-04**	**4.40E-04**	4.47E-01	**5.41E-04**	2.97E-01	**2.56E-02**
$^{13}C_2$-Gly	**6.45E-04**	**1.84E-04**	1.09E-01	**2.92E-02**	7.04E-01	4.44E-01
^{13}C-Ser	**2.24E-04**	**2.78E-05**	**3.66E-03**	**5.14E-04**	8.82E-01	**4.77E-03**
^{13}C-Gly	**4.34E-04**	**2.51E-04**	4.00E-01	1.44E-01	7.01E-01	8.38E-01
	**Q for µmole/g protein [b]**					
$^{13}C_3$-Ser	**6.92E-04**	**1.86E-02**	8.46E-01	5.26E-02	**3.79E-02**	**1.53E-02**
$^{13}C_2$-Gly	**6.83E-04**	**9.65E-03**	9.80E-01	8.71E-02	**3.47E-02**	**1.78E-02**
^{13}C-Ser	**4.71E-04**	**1.98E-02**	1.11E+00	3.40E-01	**2.86E-02**	2.27E-01
^{13}C-Gly	**4.71E-04**	9.12E-01	7.90E-01	5.30E-01	5.36E-02	**2.00E-02**
AXP ^{13}C-1'	**2.76E-03**	4.19E-01	9.40E-01	5.80E-01	7.61E-01	3.05E-01
AXP ^{13}C-2	**1.21E-03**	**6.05E-03**	8.18E-01	6.72E-01	8.84E-01	7.36E-01
AXP ^{13}C-8	**1.28E-01**	**2.36E-02**	7.45E-01	5.01E-01	7.18E-01	9.55E-01
	**Q for ^{15}N fractional enrichment [c]**					
^{15}N-Adenine	**1.24E-02**	**2.67E-03**	**7.74E-03**	**7.61E-03**	**2.63E-04**	**1.96E-05**
^{15}N-Guanine	**1.86E-02**	**1.56E-03**	**1.22E-02**	**3.96E-03**	**4.12E-04**	**1.39E-04**

[a] The Q values for the fractional distribution of ^{13}C labeled metabolites in **Fig. S7A** and [c] those of ^{15}N labeled metabolites in **Fig. S7B** were calculated from FT-MS data while those of µmole/g protein were calculated from GC-MS (for Ser and Gly) and 1D HSQC NMR (for AXP) as in **Table S1**. Bolded values denote statistical significance ($Q \leq 0.05$).

Table S6. Q values for Se agent-induced changes in fractional distribution of ^{13}C labeled isotopologues of pyrimidine nucleotides and precursor in $^{13}C_6$-glucose-treated A549 and H1299 cells.

	Q for fractional enrichment [a]					
	**A549**			**H1299**		
	Ctl vs MSA	Ctl vs SeO_3	Ctl vs SeM	Ctl vs MSA	Ctl vs SeO_3	Ctl vs SeM
$^{13}C_2$-Asp	**1.22E-03**	**7.25E-04**	7.63E-01	**2.75E-05**	2.00E-01	6.46E-01
$^{13}C_3$-Asp	**1.10E-03**	4.06E-01	**2.00E-02**	**2.95E-04**	5.28E-01	**3.85E-03**
CTP $^{13}C_{1-2}$-ring	**1.21E-03**	**5.87E-04**	**6.53E-03**	**3.33E-04**	**2.90E-02**	**6.66E-05**
CTP $^{13}C_3$-ring	**9.59E-04**	**8.40E-04**	**1.10E-02**	**2.73E-04**	3.85E-01	**2.51E-04**
UTP $^{13}C_{1-2}$-ring	2.55E-01	**3.57E-02**	**1.72E-02**	3.64E-01	1.68E-01	4.11E-01
UTP $^{13}C_3$-ring	2.29E-01	**3.40E-02**	**1.96E-02**	3.61E-01	1.50E-01	3.93E-01
	**Q for µmole/g protein** [b]					
$^{13}C_2$-Asp	**8.43E-04**	**4.28E-04**	8.30E-01	**4.35E-04**	1.05E+00	6.30E-02
$^{13}C_3$-Asp	**1.49E-05**	**3.23E-03**	9.96E-01	**4.91E-04**	1.48E+00	6.89E-01
UXP ^{13}C-1'	**2.93E-03**	**5.98E-03**	1.45E+00	9.93E-01	9.69E-00	2.67E-01
UXP ^{13}C-6	**3.76E-03**	1.35E-01	1.01E+00	**4.48E-02**	9.79E-00	9.51E-01

[a] The Q values for the fractional distribution of ^{13}C labeled metabolites in **Fig. 4A** were calculated from FT-MS data while those for µmole/g protein were calculated from GC-MS (for Asp) and 1D HSQC NMR (for UXP) as in **Table S1**. Bolded values denote statistical significance ($Q \leq 0.05$).

Table S7. Q values for Se agent-induced changes in concentrations and fractional enrichment of ^{13}C labeled isotopologues of Krebs cycle metabolites in $^{13}C_5,^{15}N_2$-Gln-treated A549 and H1299 cells.

	Q for µmole/g protein [a]					
	**A549**			**H1299**		
	Ctl vs MSA	Ctl vs SeO_3	Ctl vs SeM	Ctl vs MSA	Ctl vs SeO_3	Ctl vs SeM
$^{13}C_1$-Lactate	2.02E-01	2.81E-01	8.09E-01	5.54E-01	7.85E-01	9.55E-01
$^{13}C_2$-Citrate	**2.29E-03**	**4.88E-03**	**7.09E-03**	**2.24E-02**	**4.85E-03**	6.65E-01
$^{13}C_2$-Fumarate	6.77E-02	**4.30E-02**	1.88E-01	**1.86E-02**	**4.49E-03**	1.43E-01
$^{13}C_2$-Lactate	2.16E-01	6.23E-02	7.53E-02	7.53E-02	2.52E-01	2.70E-01
$^{13}C_2$-Malate	**1.83E-02**	**6.91E-03**	2.17E-01	2.26E-01	8.92E-02	7.14E-01
$^{13}C_3$-Citrate	**1.70E-02**	**1.14E-02**	5.28E-01	2.32E-01	8.32E-02	1.67E-01
$^{13}C_3$-Lactate	**2.34E-02**	6.11E-02	1.84E-01	5.97E-02	8.40E-02	2.59E-01
$^{13}C_4$-Asp	1.54E-01	**5.39E-03**	6.18E-02	3.89E-01	8.03E-02	2.19E-01
$^{13}C_4$-Citrate	**6.80E-03**	**5.79E-03**	1.95E-01	**2.93E-02**	**1.35E-02**	2.58E-01
$^{13}C_4$-Fumarate	**7.29E-03**	**9.02E-03**	1.03E-01	1.01E-01	**4.94E-03**	6.45E-01
$^{13}C_4$-Malate	**8.34E-03**	**1.40E-03**	1.97E-01	3.66E-01	8.19E-02	5.72E-01
$^{13}C_5$-Asp	6.43E-02	**1.63E-03**	6.16E-02	2.25E-01	7.31E-02	2.80E-01
$^{13}C_5$-Citrate	**3.38E-02**	**4.31E-02**	7.89E-02	3.74E-01	9.04E-02	2.56E-01
$^{13}C_5,^{15}N_2$-Gln	5.73E-01	1.78E-01	8.43E-01	1.60E-01	8.99E-02	6.36E-01
$^{13}C_5+^{13}C_5,^{15}N_1$-Glu	5.72E-03	1.33E-02	6.66E-02	5.65E-01	7.46E-02	6.19E-01
	**Q for fractional enrichment [b]**					
$^{13}C_1$-Lactate	7.36E-01	8.96E-01	8.40E-01	6.28E-01	4.79E-01	7.70E-01
$^{13}C_2$-Citrate	6.65E-02	7.03E-02	8.90E-02	1.60E-01	**1.83E-02**	1.92E-01
$^{13}C_2$-Fumarate	9.21E-02	5.19E-02	7.53E-02	2.01E-01	**1.57E-02**	1.14E-01
$^{13}C_2$-Lactate	2.71E-01	5.56E-02	**4.03E-02**	2.08E-01	3.09E-01	8.05E-02
$^{13}C_2$-Malate	8.13E-02	**1.74E-02**	7.47E-02	2.72E-01	8.74E-07	**4.49E-02**
$^{13}C_3$-Citrate	5.11E-01	**1.44E-02**	5.83E-01	2.92E-01	7.37E-02	2.63E-01
$^{13}C_3$-Lactate	**4.39E-02**	8.41E-01	5.35E-01	8.85E-02	**1.61E-02**	1.62E-01
$^{13}C_4$-Asp	8.60E-02	**1.14E-02**	8.02E-02	2.08E-01	**2.46E-02**	1.17E-01
$^{13}C_4$-Citrate	**3.42E-02**	**1.53E-02**	5.52E-01	4.19E-01	**2.09E-02**	6.48E-02
$^{13}C_4$-Fumarate	6.59E-02	**1.28E-02**	8.08E-01	2.72E-01	**1.55E-02**	3.25E-01
$^{13}C_4$-Malate	7.88E-02	**7.83E-03**	7.19E-02	2.86E-01	**1.62E-02**	7.21E-02
$^{13}C_5$-Asp	1.42E-01	**8.75E-03**	8.40E-02	1.75E-01	**1.36E-02**	**3.77E-02**
$^{13}C_5$-Citrate	8.76E-02	6.98E-02	6.71E-02	4.12E-01	9.95E-02	6.50E-01
$^{13}C_5,^{15}N_2$-Gln	7.08E-01	3.87E-01	2.62E-01	7.19E-01	5.85E-02	7.56E-01
$^{13}C_5+^{13}C_5,^{15}N_1$-Glu	5.88E-02	**1.79E-02**	**2.70E-02**	2.79E-01	**2.07E-02**	5.09E-02

[a,b] The Q values for the changes in ^{13}C labeling of metabolites in **Figs. 3** and **S6** were calculated from GC-MS data as in **Table S1**. Bolded values denote statistical significance ($Q \leq 0.05$).

Table S8. **Q values for Se agent-induced changes in fractional distribution of ^{13}C labeled isotopologues of pyrimidine nucleotides and precursor in $^{13}C_5$,$^{15}N_2$-Gln-treated A549 and H1299 cells.**

	Q for fractional enrichment [a]					
	A549			H1299		
	Ctl vs MSA	Ctl vs SeO_3	Ctl vs SeM	Ctl vs MSA	Ctl vs SeO_3	Ctl vs SeM
$^{13}C_2$-Asp	5.09E-02	**1.06E-02**	**3.64E-02**	**2.55E-02**	**2.11E-02**	**3.67E-02**
$^{13}C_5$-Asp	1.04E-01	**1.17E-03**	**3.36E-02**	8.73E-02	**3.62E-03**	**1.00E-02**
CTP $^{13}C_{1-2}N_x$	8.85E-02	**1.07E-02**	**1.12E-02**	5.33E-02	**2.31E-03**	**4.75E-03**
CTP $^{13}C_3N_x$	**1.65E-02**	**6.46E-04**	**5.30E-03**	6.02E-01	**1.96E-02**	**9.11E-03**
UTP $^{13}C_{1-2}N_x$	6.75E-02	**2.70E-03**	**1.42E-03**	5.52E-02	**1.89E-03**	**4.42E-03**
UTP $^{13}C_3N_x$	**4.27E-03**	**4.75E-04**	2.94E-01	6.57E-01	**9.64E-03**	**9.24E-03**
	Q for µmole/g protein [b]					
$^{13}C_2$-Asp	5.09E-02	**5.38E-03**	6.86E-02	1.54E-01	6.37E-02	1.12E-01
$^{13}C_5$-Asp	**4.29E-02**	**4.35E-04**	**4.93E-02**	1.35E-01	**4.39E-02**	4.29E-01

[a] The Q values for the fractional distribution of ^{13}C labeled metabolites in **Fig. 4B** were calculated from FT-MS data while those for µmole/g protein were calculated from GC-MS (for Asp) as in **Table S1**. Bolded values denote statistical significance (Q ≤ 0.05).

Table S9. **Q values for Se agent-induced changes of protein expression in $^{13}C_6$-glucose-treated A549 and H1299 cells**.

	Q for protein expression [a]					
	**A549**			**H1299**		
	Ctl vs MSA	Ctl vs SeO_3	Ctl vs SeM	Ctl vs MSA	Ctl vs SeO_3	Ctl vs SeM
NDUFS1	**1.87E-02**	**6.70E-04**	6.53E-02	2.06E-01	8.75E-01	2.06E-01
MTAPT8	**7.25E-03**	**4.54E-04**	1.16E-01	3.85E-01	5.28E-01	1.90E-01
TFAM	**3.18E-02**	**8.95E-04**	1.17E-01	1.81E-01	8.78E-01	2.38E-01
PC	2.75E-01	**8.25E-04**	1.63E-01	3.98E-01	6.62E-01	1.96E-01
GLS1 (KGA/GAC)	1.93E-00	**9.13E-04**	2.28E-01	1.65E-01	8.33E-01	1.69E-01
GAC	2.16E-01	**3.85E-04**	1.88E-01	1.30E-01	**8.72E-04**	6.39E-01
GLS2	**2.74E-02**	**3.77E-04**	1.05E-01	**3.61E-02**	7.45E-01	**8.38E-03**
CAD	**4.99E-02**	**2.14E-03**	1.20E-01	4.09E-01	1.17E-01	1.52E-01
PHGDH	**2.39E-02**	**1.06E-03**	1.89E-01	1.82E-01	8.47E-01	6.68E-02
PSAT1	**1.69E-02**	**1.04E-03**	1.26E-01	5.90E-01	4.66E-01	1.85E-01
SHMT2	7.85E-01	**1.95E-03**	3.90E-01	9.28E-01	9.02E-01	5.76E-01
SHMT1	**4.04E-03**	**2.08E-04**	1.26E-01	1.82E-01	8.80E-01	5.25E-02
GLDC	**4.33E-03**	**4.32E-04**	**4.18E-02**	1.27E-01	**4.47E-02**	5.50E-01
ME1	**1.56E-02**	**3.65E-04**	4.15E-01	1.27E-01	7.33E-01	6.18E-01
ME2	**1.74E-02**	**1.82E-05**	5.15E-02	3.33E-01	7.95E-01	2.07E-01
ACLY	**8.23E-03**	**4.10E-05**	**1.65E-02**	5.74E-01	5.39E-01	6.24E-01
FASN	**8.01E-03**	**1.18E-04**	2.66E-01	7.15E-01	7.98E-01	9.46E-01

The Q values for changes in protein expression were calculated from RPPA data in **Fig. S5** as in **Table S1**. Bolded values denote statistical significance ($Q \leq 0.05$).

Table S10. **Q values for PC KD-induced changes of ROS production, cell cycle, and cell death in A549 and H1299 cells**.

	**Q for ROS production** [a]			
	**A549**		**H1299**	
	Ctl vs PC54	Ctl vs PC55	Ctl vs PC54	Ctl vs PC55
Day 8	7.64E-01	1.55E-04	2.26E-05	6.70E-04
Day 9	3.40E-03	1.71E-04	5.37E-08	8.10E-04
	Q for Cell cycle [b]			
G2/M	1.04E+00	4.44E-06	4.25E-04	7.80E-05
S	8.46E-01	3.43E-04	2.52E-03	2.33E-04
G1	1.13E-05	2.43E-05	2.72E-03	4.71E-01
	**Q for Apoptosis/Necrosis** [c]			
EA	4.12E-04	7.46E-04	3.47E-05	7.69E-05
Viable	1.85E-03	4.18E-05	1.03E-03	8.99E-05
LA/Nec	4.61E-03	6.76E-04	2.55E-03	2.04E-04
Nec	4.98E-03	1.19E-03	9.77E-04	8.56E-05

The Q values for changes in ROS production, cell cycle parameters, and apoptosis/necrosis in PC KD cells were calculated from flow cytometry data in **Fig. S8A**-**B** as in **Table S1**. Bolded values denote statistical significance ($Q \leq 0.05$).

Table S11. Q values for GLS1 KD-induced changes of ROS production, cell cycle, and cell death in A549 and H1299 cells.

	Q for ROS production [a]			
	**A549**		**H1299**	
	EV vs GLS35	EV vs GLS36	EV vs GLS35	EV vs GLS36
Day 8	**3.66E-05**	**2.42E-03**	**2.49E-05**	**1.81E-04**
Day 9	**1.38E-05**	**4.84E-03**	**5.74E-04**	5.93E-02
	**Q for Cell cycle [b]**			
G2/M	**8.31E-04**	**2.49E-03**	**3.65E-05**	**1.08E-03**
S	**1.27E-04**	**2.44E-04**	**3.89E-04**	**9.32E-04**
G1	**4.82E-05**	**1.36E-04**	**1.99E-03**	**5.00E-02**
	**Q for Apoptosis/Necrosis [c]**			
EA	**3.89E-02**	3.84E-01	2.21E-01	3.03E-01
Viable	**4.71E-03**	2.09E-01	**9.61E-05**	**1.44E-03**
LA/Nec	**1.61E-05**	**5.15E-03**	**6.00E-04**	**1.31E-03**
Nec	**2.00E-04**	1.14E-01	**1.19E-04**	**1.43E-03**

The Q values for changes in ROS production, cell cycle parameters, and apoptosis/necrosis in GLS1 KD cells were calculated from flow cytometry data in **Fig. S8C-D** as in **Table S1**. Bolded values denote statistical significance ($Q \leq 0.05$).

Table S12. **Q values for Se agent-induced changes of lipid metabolism in $^{13}C_6$-glucose-treated A549 and H1299 cells**.

	Q for ^{13}C Fractional enrichment in $^{13}C_6$-glucose study [a]					
	A549			H1299		
	Ctl vs MSA	Ctl vs SeO_3	Ctl vs SeM	Ctl vs MSA	Ctl vs SeO_3	Ctl vs SeM
$^{13}C_2$-Citrate	**4.42E-06**	**4.45E-04**	**7.88E-06**	**4.45E-02**	8.25E-02	**1.05E-02**
$^{13}C_3$-GlyOH3P	**3.19E-03**	**3.98E-03**	**1.18E-05**	2.27E-01	9.29E-01	**4.74E-03**
^{13}C-PC-FA	**8.73E-04**	**1.04E-05**	**4.29E-05**	**5.48E-03**	3.87E-01	**2.39E-02**
^{13}C-PC-GlyOH	**1.45E-05**	**2.03E-07**	**3.28E-04**	**2.32E-02**	7.81E-01	**8.05E-03**
^{13}C-PI-FA	**1.24E-05**	**1.25E-07**	**4.45E-04**	**7.11E-04**	1.93E-01	**9.93E-03**
^{13}C-PI-GlyOH	**3.36E-06**	**1.06E-05**	**3.98E-03**	**9.04E-05**	**9.21E-03**	**4.91E-04**
	**Q for ^{13}C µmole/g protein in $^{13}C_6$-glucose study [b]**					
$^{13}C_2$-Citrate	**7.26E-05**	**3.66E-05**	2.54E-01	**2.51E-05**	3.22E-01	**1.22E-02**
$^{13}C_3$-GlyOH3P	**1.01E-02**	**2.28E-05**	4.26E-01	1.24E-01	3.64E-01	**1.39E-02**
	**Q for ^{13}C Fractional enrichment in $^{13}C_5$,$^{15}N_2$-Gln study [c]**					
$^{13}C_2$-Citrate	5.32E-02	1.12E-01	7.12E-02	1.92E-01	**3.66E-02**	7.68E-01
$^{13}C_3$-GlyOH3P	7.34E-02	9.91E-01	6.47E-01	1.03E-01	8.44E-01	6.86E-01
^{13}C-PC_{even}	**3.99E-02**	**1.31E-02**	**4.74E-02**	4.97E-01	1.14E+00	8.44E-01
^{13}C-PC_{odd}	**4.51E-02**	3.29E-01	5.24E-01	5.12E-01	7.70E-01	7.62E-01
^{13}C-SM_{even}	**1.28E-02**	**2.69E-03**	2.47E-01	8.45E-01	6.34E-01	6.34E-01
^{13}C-SM_{odd}	4.85E-01	6.46E-01	5.74E-01	6.34E-01	5.07E-01	5.07E-01
	**Q for ^{13}C µmole/g protein in $^{13}C_5$,$^{15}N_2$-Gln study [d]**					
$^{13}C_2$-Citrate	**3.05E-04**	**1.95E-03**	**9.45E-04**	**5.97E-03**	**1.29E-03**	1.24E+00
$^{13}C_3$-GlyOH3P	3.54E-01	4.40E-01	2.60E-01	9.34E-02	9.47E-01	7.40E-01

[a-d] The Q values for the changes in ^{13}C labeling of lipid and precursor metabolites in **Figs. 5A** and **5B** were calculated from GC-MS and FT-MS data as in **Table S1**. Bolded values denote statistical significance ($Q \leq 0.05$).

Reference

110. Benjamini, Y. and Y. Hochberg, *Controlling the False Discovery Rate: A Practical and Powerful Approach to Multiple Testing.* Journal of the Royal Statistical Society. Series B (Methodological), 1995. **57**(1): 289-300.
